# Biological and physiochemical studies of electrospun polylactid/polyhydroxyoctanoate PLA/P(3HO) scaffolds for tissue engineering applications†

**DOI:** 10.1039/d3ra03021k

**Published:** 2023-08-11

**Authors:** Daria Solarz, Tomasz Witko, Robert Karcz, Ivana Malagurski, Marijana Ponjavic, Steva Levic, Aleksandra Nesic, Maciej Guzik, Sanja Savic, Jasmina Nikodinovic-Runic

**Affiliations:** a Jerzy Haber Institute of Catalysis and Surface Chemistry, Polish Academy of Sciences Niezapominajek 8 30-239 Krakow Poland tomasz.witko@ikifp.edu.pl +48 507196 866; b Faculty of Physics, Astronomy and Applied Computer Science, Jagiellonian University Lojasiewicza 11 30-348 Krakow Poland; c Department of Product Technology and Ecology, Krakow University of Economics Rakowicka 27 31-510 Kraków Poland; d Institute of Molecular Genetics and Genetic Engineering, University of Belgrade 11042 Belgrade Serbia jasmina.nikodinovic@imgge.bg.ac.rs +381 11 397 60 34; e Faculty of Agriculture, University of Belgrade 11081 Belgrade Serbia; f Faculty of Technology 21000 Novi Sad Serbia; g University of Belgrade, Institute of Chemistry, Technology and Metallurgy, Center of Excellence in Environmental Chemistry and Engineering Njegoseva 12 11000 Belgrade Serbia

## Abstract

Polyhydroxyoctanoate, as a biocompatible and biodegradable biopolymer, represents an ideal candidate for biomedical applications. However, physical properties make it unsuitable for electrospinning, currently the most widely used technique for fabrication of fibrous scaffolds. To overcome this, it was blended with polylactic acid and polymer blend fibrous biomaterials were produced by electrospinning. The obtained PLA/PHO fibers were cylindrical, smaller in size, more hydrophilic and had a higher degree of biopolymer crystallinity and more favorable mechanical properties in comparison to the pure PLA sample. Cytotoxicity evaluation with human lung fibroblasts (MRC5 cells) combined with confocal microscopy were used to visualize mouse embryonic fibroblasts (MEF 3T3 cell line) migration and distribution showed that PLA/PHO samples support exceptional cell adhesion and viability, indicating excellent biocompatibility. The obtained results suggest that PLA/PHO fibrous biomaterials can be potentially used as biocompatible, biomimetic scaffolds for tissue engineering applications.

## Abstract 2

The work investigates fabrication and physicochemical and biological evaluation of electrospun polyhydroxyoctanoate and polylactic acid blend fibers. The results suggest that the obtained fibrous matrices can be used as biomimetic biomaterials.

## Graphical abstract

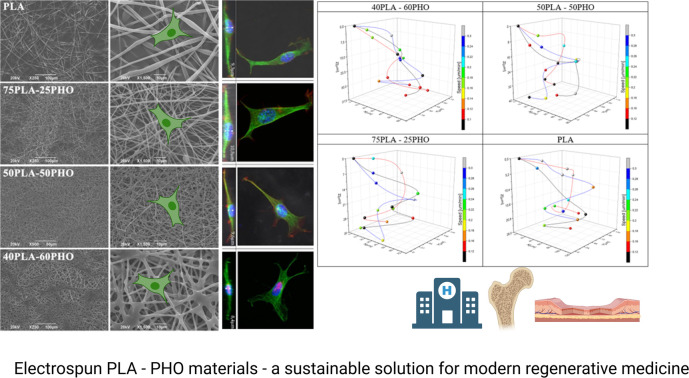

## 1. Introduction

Medium chain length polyhydroxyalkanoates (mcl-PHAs) are elastomeric polyesters produced by bacteria under nutrient limiting conditions^1^ and sustainably from a variety of waste sources.^2^ Tunable mechanical properties coupled with low crystallinity (due to the loosely and irregularly packed side chains within the biopolymer molecule) provide good biocompatibility^3^ and controllable degradation rate,^4^ making mcl-PHAs an excellent candidate for biomedical application. Additionally, some mcl-PHAs monomers and their derivatives are bioactive and can be used as antimicrobial and anticancer drugs.^5^ The abundance of amorphous regions also means that mcl-PHAs are characterized by low glass transition and melting temperature and poor tensile strength, which limits their potential for processing into scaffolds that imitate the nanofibrous structure of the extracellular matrix. A simple yet efficient way to produce highly porous, fibrous, biomimetic scaffolds is electrospinning. During this process, electrostatic force stretches polymer solution droplets into fine jets, which subsequently solidify into fibers upon solvent evaporation. Unfortunately, mcl-PHAs are not suitable for electrospinning because the presence of relatively bulky side chains interferes with the biopolymer entanglement necessary for fiber formation.^6^ To electrospin mcl-PHA into fibers, mcl-PHAs must be blended with another electrospinable polymer, like polylactic acid (PLA), which acts as a carrier phase. PLA is a synthetic biocompatible biopolymer obtained from corn starch or sugar cane through a chemo-biotechnological process. This aliphatic polyester is the subject of many biomedical studies and has already been approved by the Food and Drug Administration (FDA) and European Medicines Agency (EMA) because it is biocompatible, biodegradable and has properties that are suitable for different kinds of processing, especially electrospinning.^7^ So far, various electrospun PLA biomaterials have been extensively studied as wound dressings,^8^ scaffolds^9^ or drug delivery systems.^10^ When compared to PHAs, PLA exhibits certain limitations regarding biomedical application. First, PLA lacks structural diversity and hence the possibility to modulate the properties to specific applications. Second, the degradation products of PLA are more acidic in comparison to PHAs. In addition to this, being produced from the food source, negatively reflects on PLA sustainability.

Considering this, combining PLA and PHO will not only improve the processing potential of PHO, but will also improve the overall biocompatibility of these PLA-containing biomaterials. This methodological approach will allow for mcl-PHAs to be increasingly utilized for high-end applications, as obtained biomaterials can be used as biocompatible, biodegradable, and sustainable biomaterials for drug delivery or tissue engineering. The presence of elastomeric, biocompatible constituent within a fibrillar scaffold that mimics extracellular matrix will improve properties and overall biocompatibility and in turn support adhesion and migration of fibroblasts which is a prerequisite for cellular migration and synthetic activity. In addition, the fabrication procedure offers control over porosity and mechanical properties of the biomaterials leading to personalized medical treatments.

The mechanical, physicochemical, and biological properties of the surrounding influence the behavior and morphology of living cells.^11^ Cells cultured on materials characterized with complex dimensional structure undergo numerous interactions mediated by adhesive cellular structures. Fibrillar material structure influences cell movement directionality.^12^ Cell spatial penetration inside the substrate occurs despite the limitations of simple diffusion caused by the density and distribution of polymer fibers. Advanced techniques such as fluorescent confocal microscopy enabled us to perform a full biological material characterization to explore their biomedical potential.

The aim of this study is to investigate whether PLA can be used as a carrier polymer to enable electrospinning of mcl-PHAs (PHO) and produce polymer blend PLA/PHO fibers as sustainable, biocompatible, biomimetic biomaterials. The effect of PHO addition on the obtained electrospun matrices properties (fiber diameter, morphology, hydrophilicity, biopolymer degree of crystallinity and mechanical characteristics) and biocompatibility (cytotoxicity and cell migration studies) was evaluated. By examining the interaction of mammalian cells (mouse embryonic fibroblasts) with the internal structure of the material, the impact of polymers on the elements of the cytoskeleton and the extensive analysis of 3D cell migration in materials, it is possible to initially determine the usefulness of the tested composites in medicine and biomedical sciences.

## 2. Materials and methods

### 2.1. Materials

Precursors used for preparation of electrospinning solution were polylactic acid (PLA), PHO, dichloromethane and dimethylformamide as solvents. PHO was produced as in our previous study using *Pseudomonas putida* KT2440 strain and octanoic acid as substrate.^13^ This polymer was composed of 91 mol% (*R*)-3-hydroxyoctanoic acid, 7 mol% (*R*)-3-hydroxyhexanoic acid, and below 2 mol% in total of other (*R*)-3-hydroxylated fatty acids.^13^

### 2.2. Electrospinning procedure

#### 2.2.1. Preparation of electrospinning solutions

Different polymer solutions were prepared using dichloromethane–dimethylformamide mixture (ratio 3 : 2, v/v) as a solvent. Prior to electrospinning, all solutions were mixed on a magnetic stirrer at room temperature for 24 h. Compositions of the samples are summarized in Table 1.

**Table 1:** Sample codes and corresponding compositions

Sample	PLA^a^ [%]	PHO [%]	Polymer concentration^b^ [wt%]
PLA	100	0	10
75PLA-25PHO	75	25	12
50PLA-50PHO	50	50	12
40PLA-60PHO	40	60	15
25PLA-75PHO	25	75	15
25PLA-75PHO	25	75	20

a Fractions of different constituents relative to the total solids weight.

b Total polymer concentration in electrospinning solution.

Electrospinning was carried out at room temperature at a lab scale electrospinning machine Fluidnatek LE-10 (Bioinicia S.L., Valencia, Spain). The polymer solution was continuously pumped at a constant flow rate through a positively charged, stainless steel needle and spun by electrostatic force onto a grounded collector. The process parameters were as follows: feed rate 2.5 mL h^−1^, inner diameter of the needle 0.8 mm, distance between the needle tip and the collector 10 cm and the applied voltage of 10–12 kV for all samples.

### 2.3. Characterization of the electrospun PLA/PHO biomaterials

#### 2.3.1. Scanning electron microscopy (SEM)

SEM imaging was done using a JEOL JSM-6390LV SEM (JEOL USA Inc., Peabody, USA), operated at 20 keV. Prior to analysis, samples were coated with a conducting layer of gold. ImageJ program (National Institutes of Health, Bethesda, MD, USA) was used to determine the average fiber diameter. By using ImageJ software program based on a threshold technique which divides pixels within the target range of intensity values^14–16^ it was also feasible to calculated average pore size and the porosity area (%).

#### 2.3.2. Water contact angle (WCA)

Wettability of the samples was examined by measuring water contact angle with an Ossila optical goniometer (Sheffield, England). Using a syringe, a drop of distilled water was placed onto the sample surface and, following the initial contact between water and the sample, the equilibrium water contact angle was determined as the mean value of the corresponding left and right contact angles. For each sample, the measurements were conducted at three different spots at room temperature.

#### 2.3.3. Fourier transform infrared spectroscopy (FTIR)

FTIR was conducted using a FTIR IRAffinity-1 spectrometer (Shimadzu, Kyoto, Japan) at room temperature using the attenuated total reflectance (ATR) technique. Spectral range was 4000–600 cm^−1^ and resolution of 4 cm^−1^.

#### 2.3.4. Differential scanning calorimetry analysis (DSC)

Differential Scanning Calorimetry (DSC) measurements were performed using a Shimadzu – DSC-60 Plus under a nitrogen purge gas flow of 50 mL min^−1^. Indium was used to calibrate the calorimeter in temperature and energy. The measurements were performed in the temperature range from 25 to 250 °C, at a heating rate of 10 °C min^−1^. Sample weight was limited to 5.0 ± 0.5 mg. Each sample was tested three times to confirm repeatability of measurements. For the determination of melting temperature, *T*_m_, glassing temperature, *T*_g_, temperature of crystallization, *T*_cc_, as well as the enthalpies, (Δ*H*_m_, Δ*H*_cc_) obtained from DSC, TRIOS Software TA Universal Data Analysis was used. The obtained melting enthalpies were determined from the area under the endotherms, while the degree of crystallinity, *X*_c_, was deduced from the calculated melting enthalpy, Δ*H*_m_, and melting enthalpy od 100% crystalline PLA, 
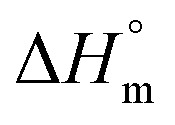
, (93.6 J g^−1^)^17^ normalized by the PLA amount (*w*) in each sample following the eqn (1):1
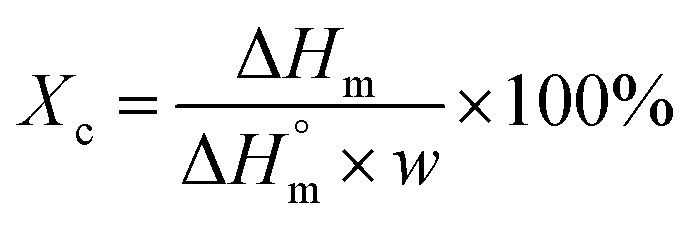


#### 2.3.5. Thermogravimetric analysis (TGA)

TGA measurements were performed using a TA Instruments SDT Q600. The samples mass in range of 5–7 mg was ramped from ambient temperature to 700 °C in an alumina crucible at a heating rate of 20 °C min^−1^. All the experiments were carried out in a nitrogen atmosphere with a flow of 20 mL min^−1^ to avoid thermoxidative degradation.

#### 2.3.6. X-ray diffraction scattering analysis (XRD)

The XRD analysis was performed to investigate the crystallography patterns of electrospun materials. XRD measurements were collected using a Bruker D2 Phaser diffractometer (Bruker, Billerica, USA) with a copper anode with Kα radiation of 1.54 Å, an electron beam energy of 30 kV, and 10 mA intensity, in the range of *2θ* from 0 to 50°.

#### 2.3.7. Mechanical analysis

The tensile mechanical properties of the investigated samples were evaluated by using Shimadzu Autograph AGS-X servo-hydraulic testing machine, equipped with a 1 kN load cell at ambient temperature and a tensile extender speed of 2 mm min^−1^. Mechanical properties including tensile modulus, ultimate tensile strength, and elongation at break were evaluated for both PLA and PLA/PHO fibers. Five measurements were performed for each sample and the average values were presented (±SD).

#### 2.3.8. Biocompatibility evaluation

##### 2.3.8.1. Cytotoxicity

Cytotoxicity evaluation was performed according to previously described protocol^18^ using MRC5 cells (human lung fibroblasts obtained from ATCC). In brief, monolayer cell cultures were treated with 50%, 25% and 12.5% (v/v) of filtered biomaterial extract and incubated for 48 h. Cell proliferation was determined using standard MTT assay.

##### 2.3.8.2. Cellular studies

Cellular studies on different substrates was conducted using mouse embryonic fibroblasts MEF 3T3 cell line and procedure described by Witko *et al.*^8^ Mouse embryonic fibroblasts MEF 3T3 cells were grown in plastic culture flasks under sterile conditions in an incubator (Thermo Fisher Scientific DH Series, Waltham, USA) maintaining constant environment (37 °C, 5% CO_2_). The culture medium used was DMEM (Dulbecco's Modified Eagle Medium) supplemented with 10% fetal bovine serum (FBS) and 1% antibiotics (penicillin and streptomycin, Sigma-Aldrich® Poznan, Poland). The cell culture was split using a standard passage procedure when the confluence reached about 80%. Cells used in the study were after third, but not beyond ninth passage.^19^ All measurements and microscopic observations were performed in fluorescent and confocal modes combined with pseudo wide field channel (T-PMT) on a Zeiss Axio Observer Z.1 microscope with LSM 710 confocal module. The image collection, processing and analysis were performed using Zeiss ZENBlack version 8.1.0.484, PALMRobo V 4.6.0.4 software.

To stain actin fibers a dye solution containing rhodamine-labeled phalloidin (Invitrogen Waltham, USA) was used. Nucleus staining was performed with use of DAPI dye (Thermo Fisher Scientific, Waltham, USA). Monoclonal Anti-α-Tubulin mouse antibody (Invitrogen, Waltham, USA) was used in combination with Goat anti-Mouse IgG secondary antibody with Alexa Fluor488 fluorophore (Thermo Fisher Scientific, Waltham, USA) was applied for α-tubulin staining (microtubular network). Light protected samples were left in the refrigerator until the imaging could be performed.

##### 2.3.8.3. Migration analysis

**Fig. 1:**
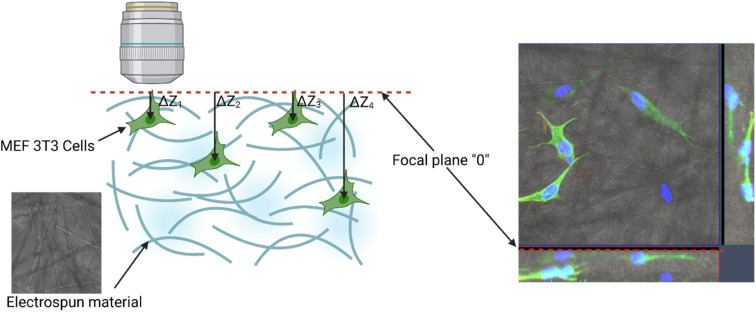
Experimental setup for cell 3D tracking and further migration analysis.

Imaging was made using an oil immersion objective with a magnification of 40× and a numerical aperture (NA) of 1.4. Cell Tracker v 1.0 Software with automatic vignetting and alignment, along with a semi-automated algorithm, was used to precisely track all cells in the field of view. For the identification of individual cells, a dynamic interpolation with histogram matching was set up. The tracking software algorithms were further optimized by using intravital staining of the cell nucleus with the NucBlue reagent from Thermo Fisher Scientific. This reagent, based on Hoechst 33342, is a commonly used cell-permeant nuclear counterstain that emits blue fluorescence upon binding to DNA. The measurement of the location of individual cells in 3 dimensions (X, Y, Z) was used to precisely determine all relevant migration parameters. The method used is an extension of the one described by Witko *et al.*^8^, but in this case the “*Z*” direction was additionally taken into account in the analysis, illustrating the penetration of cells inside the materials internal 3D structure. The 3D migration analysis protocol is illustrated in Fig. 1.

### 2.4. Statistical analysis

GraphPad Prism 9.4.1 software (GraphPad Prism 9.4.1, La Jolla, CA, USA) was used to perform a one-way analysis of variance (ANOVA), followed by Tukey's multiple comparisons test. The results are presented as mean ± standard deviations (SD). The difference was statistically significant at *p* ≤ 0.05.

## 3. Results and discussion

### 3.1. PLA/PHO fibers size and morphology

**Fig. 2:**
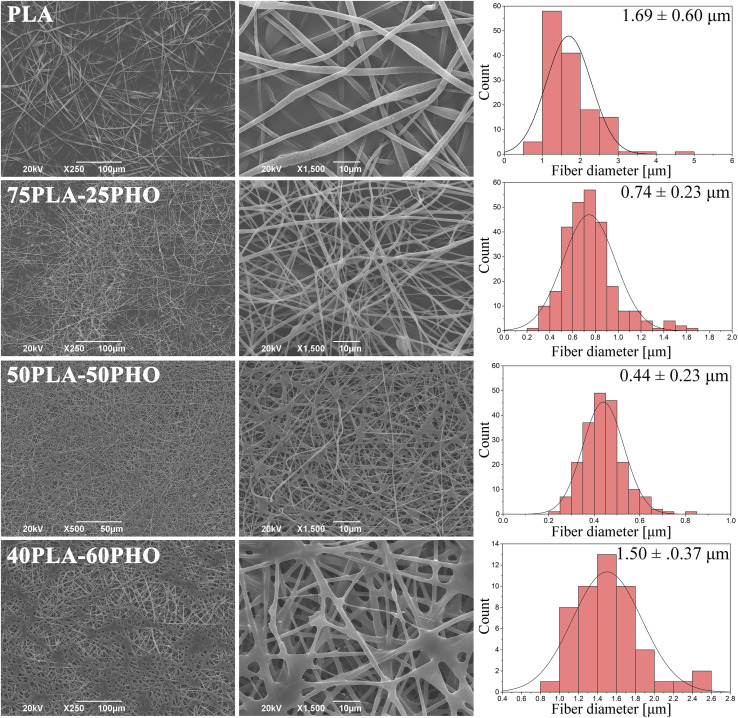
SEM micrographs of the neat PLA and PLA/PHO electrospun fibers with increasing PHO content; corresponding diameter distribution diagrams are given in the right column.

PHO was successfully electrospun by blending with PLA. Samples in the form of nonwoven fibrous mats were obtained using formulations with 75/25, 50/50 and 40/60 PLA/PHO w/w ratios. The sample with the highest PHO content (25PLA/75PHO) could not be electrospun even at the higher total polymer concentration (20 wt%). Micrographs and corresponding diameter distribution histograms of the obtained samples are presented in Fig. 2.

Fibers' morphology and diameter were significantly influenced by the presence of PHO and its content. Pure PLA fibers were almost ribbon-like, with the highest diameter (1.6957 ± 0.6059 μm) and their surface appeared porous. In contrast to this, PLA/PHO fibers were more cylindrical, without pores on the surface and increase in the PHO content has led to the reduced values of the average fiber dimeter. However, some morphology defects were also observed: (i) beads and (ii) some fibers welded/fused together at their crossing points. As the content of PHO was further increased (40PLA-60PHO), the fusion points were more pronounced, and the diameter of the fibers has increased again.

Ribbon-like morphology and big diameter of the pure PLA fibers might have been caused by the high concentration of the electrospinning solution and/or short distance between tip of the needle and the collector. In both cases, the jet did not have time to extend, stretch into thin fibers and solidify before reaching the collector. When these partially solidified fibers were deposited, their morphology was flattened due to the impact with the collector.^20^ Porous structure can be explained by phase separation during electrospinning which was probably caused by ambient conditions under which the electrospinning process was conducted (*i.e.* high relative humidity). High humidity affects fiber morphology in two ways: (i) it inhibits solvent evaporation and subsequently solidification (leading to higher diameters) and (ii) water molecules can condensate on the surface of a jet, penetrate fiber interior causing phase separation between polymer and water. After solvent and water evaporation, pores are formed in the region where water was present because these regions were depleted of polymer.^21,22^

The addition of PHO has affected polymer blend PLA/PHO fibers size and morphology, which can be explained by the changes in the electrospinning solution properties in the presence of PHO. The latter molecules have bulky side chains which has interfered with the polymer chain entanglement leading subsequently to the lower viscosity and surface tension of the PLA/PHO electrospinning solutions. This has reduced jet resistance to the extension and resulted in smaller diameters of the PLA/PHO fibers. Sample 75PLA-25PHO exhibited smaller diameter fibers, but some beaded structures were present suggesting, probably, inhomogeneous dispersion of PHO within the electrospinning solution. In this case, PHO-rich jet would have low viscosity which would result in insufficient stretching and beads formation. The most uniform fibers, with the narrowest size distribution and smallest fiber diameter, were in 50PLA-50PHO sample. Viscosity of the 50PLA-50PHO electrospinning solution was in the optimal range, however, slow crystallization of PHO,^23^ which was present in substantial amount, caused fusions at multiple contact, crossing points between adjacent layers of semi-solidified fibers. If the polymer crystallizes slowly, fibers are not fully solidified upon deposition which leads to welding or fusion at contact, crossing points. This can in turn affect electrospun mat porosity and mechanical properties. When the amount of PHO was further increased (40PLA-60PHO sample), the fiber diameter increased and fused structures became more pronounced, forming film-like regions. Deposited fibers could not retain their shape and fused together because high PHO content inhibited crystallization of the polymer blend. Similar findings were reported for the mcl-PHA/scl-PHA electrospun blends.^6^ Due to this, electrospinning of the sample with the highest PHO content (25PLA-75PHO) resulted in sticky mats, which completely lost their fibrous morphology (data not shown).

Regarding the pore size and porosity estimated by ImageJ software, fiber diameter determines the average pore size as was reported in the literature^24^ and follows the trend observed for diameter size distribution. Therefore, the neat PLA sample with the highest fiber diameter had an average pore size of 12.9 μm, while PLA/PHO fibers were characterized with the lower values of estimated pore size, with the lowest calculated for the 50PLA-50PHO fiber which had the smallest fiber diameter in the series (Table 4). It is important to mention that ImageJ program measures the pore size from the 2D SEM images which does not take into account pore distribution throughout the samples depth, but the collected results were important to confirm the trend between the biomaterials of different compositions and different average fiber diameter.

### 3.2. PLA/PHO fibers wettability

Wettability of the obtained samples was evaluated by measuring water contact angle. The neat PLA sample was hydrophobic with the value of water contact angle around 131° (Fig. S1†) which is in accordance with the literature.^25^ The addition of PHO has caused a decrease in water contact angle for all polymer blend formulations in comparison to the pure PLA sample. This improvement in wettability of the polymer blend fibrous biomaterials is caused by the change in the sample composition and surface morphology. Blending with PHO which possesses more polar functional groups (from the polymer chain ends) available to form hydrogen bonds with water resulted in the decrease of WCA values, but still WCA was in the range describing hydrophobic material (WCA above 90°). Solvent cast PHO films have WCA of ∼100°,^26^ which is significantly smaller than the WCA of the pure PLA sample. PLA/PHO fibers were also smaller in size, had smaller interfibrillar spaces and exhibited patches of film-like fused regions affecting in turn surface properties of the obtained PLA/PHO electrospun matrices and decreasing the overall WCA.

### 3.3. FTIR analysis

**Fig. 3:**
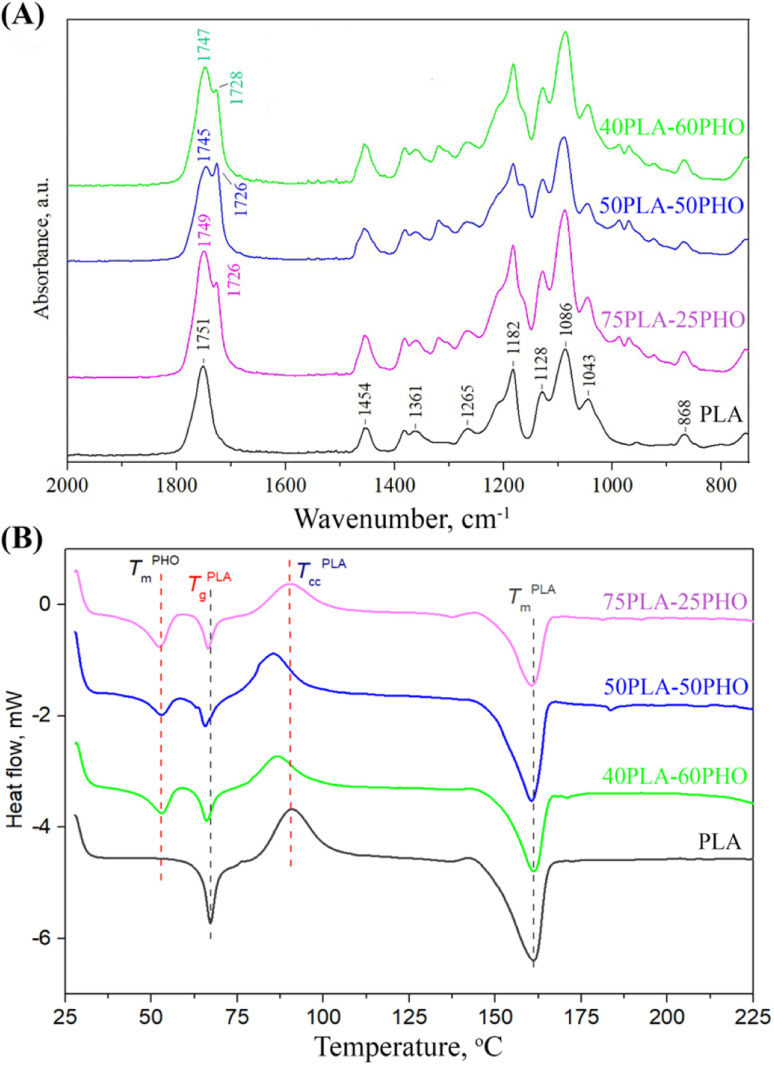
Characterization of the neat PLA and PLA/PHO fibers with increasing PHO content: (A) FTIR spectra; (B) DSC thermograms.

To investigate the existence and type of interactions between constituents of the polymer blend fibers, FTIR analysis was conducted. The obtained FTIR spectra are presented in Fig. 3A.

**Fig. 4:**
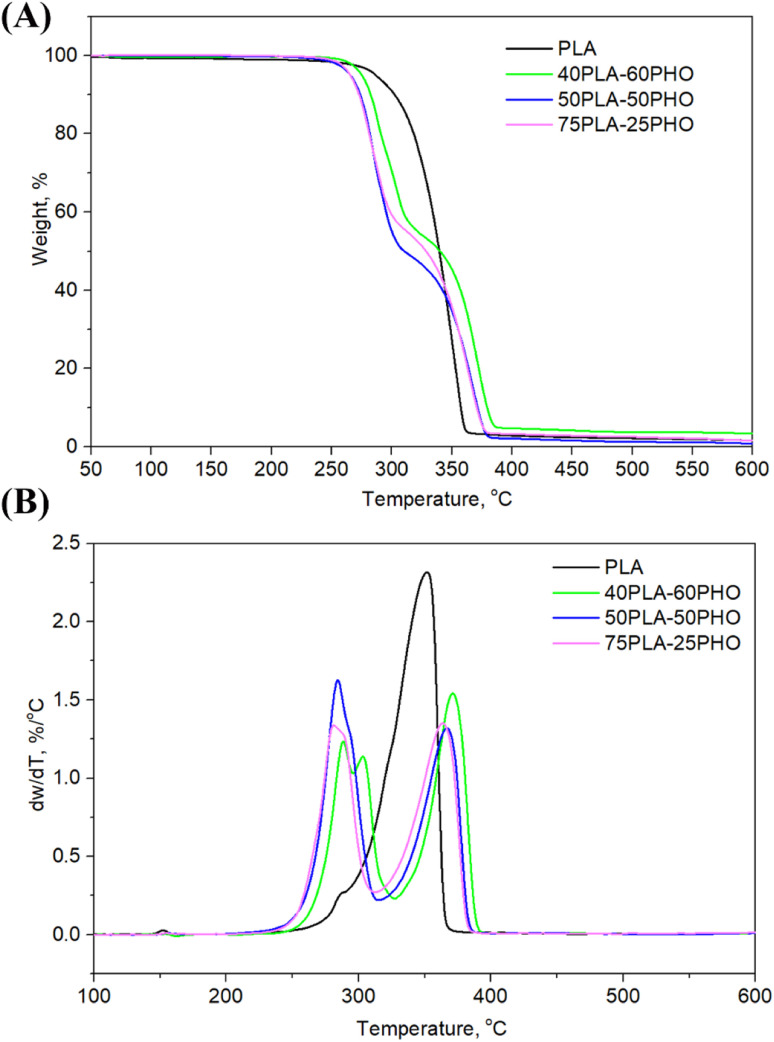
(A) TGA and (B) DTGA curves of the investigated fibers.

The spectrum of the neat PLA fibers has exhibited the characteristic peaks for amorphous PLA: the prominent peak at 1751 cm^−1^ (stretching vibrations of C=O); bands at 1454 cm^−1^ and 1361 cm^−1^ (asymmetric and symmetric –CH_3_ vibrations, respectively); at 1265 cm^−1^ (C=O bending); at 1182 cm^−1^ (–C–O–C– stretching); at 1128 cm^−1^ and 1086 cm^−1^ (C–O stretching) and at 868 cm^−1^ (–C–C– stretching in the PLA amorphous phase). The addition of PHO has introduced another peak at 1726 cm^−1^, which is the main band in the PHO spectra and is assigned to the ester carbonyl group stretching in the PHO molecule. The position of the PHO ester C=O band shows that the carbonyl group is involved in the weak hydrogen bonding with the PHO side-chains, indicating that PHO constituent is in semi-crystalline state. The changes in PLA/PHO fiber composition have resulted in the changes in the corresponding FTIR spectra, predominantly in the region of carbonyl stretching. The increase in the PHO fiber content has caused a shift in the PLA C=O peak from 1751 to 1749, 1745 and 1747 cm^−1^ for PLA, 75PLA-25PHO, 50PLA-50PHO and 40PLA-60PHO sample, respectively (Fig. 4), indicating the formation of interactions between characteristic functional groups of PLA and PHO molecules.

### 3.4. DSC analysis

DSC analysis was performed to get an insight into the thermal properties of the obtained PLA and PLA/PHO fibers. The obtained thermograms were presented in Fig. 3B, where in the temperature range of 50 °C to 170 °C, following inflection points appeared: (a) glass transition temperature (*T*_g_) near 65 °C of PLA, (b) a crystallization peak (*T*_cc_) of PLA at about 90 °C, and (c) melting temperature peaks (*T*_m_) of both PLA and PHO near 160 °C and 53 °C, respectively.

The neat PLA fiber showed a single melting peak at 161.2 °C, while the cold crystallization peak appeared at 90.8 °C, as was expected.^27^ These values, as well as those defined for the PLA/PHO fibers including characteristic enthalpies, are listed in Table 2. From the Fig. 3B and Table 2, it could be concluded that both *T*_m_ values of PLA and PHO remained unchanged after the blending and fiber processing. In contrast to this, *T*_g_ values of PLA have remarkably changed after blending with PHO confirming that mixing with PHO had an impact on the chain mobility of PLA. When the PHO was blended in higher amount, 40/60 and 50/50, this effect was more prominent and the decrease in *T*_g_ value was clearly visible. Further, from Fig. 3B, regarding the cold crystallization temperatures, it could be noticed that neat PLA indicated cold crystallization peak at 90.8 °C, while *T*_cc_ temperatures of PLA/PHO fibers shifted to lower values clearly demonstrating crystallization promoting effect of PHO. Moreover, from Table 2, the remarkable change in *T*_cc_ values was obvious, when the value of 90.8 °C detected for neat PLA decreased to 85.5 °C measured for 50PLA-50PHO fiber. Consecutively, calculated melting enthalpies of PLA/PHO fibers were lower compared to neat PLA further reflecting to the degree of crystallinity values, *X*_c_. Hence, enhancing effect of PHO to PLA crystallization resulted in the higher *X*_c_ values of PLA/PHO fibers with greater amount of PHO, 40/60 and 50/50. While the crystallinity of 75PLA-25PHO was close to those calculated for neat PLA (15.4% compared to 19.8%), *X*_c_ values of 40PLA-60PHO and 50PLA-50PHO appeared dramatically higher, 36.1% and 30.3%, respectively. The highest crystallinity of the 50PLA-50PHO fibers can be additionally attributed to the smallest, uniform fibers size (0.32 μm) defined by SEM analysis, which provided more compact, highly ordered material structure. Finally, by blending PLA and PHO polymers and electrospinning, PLA/PHO fiber mats with enhanced and tunable thermal properties can be easily produced.

**Table 2:** DSC analysis results

Sample	*T* ^PLA^_g_, °C	*T* ^PHO^_m_, °C	*T* ^PLA^_cc_, °C	*T* ^PLA^_m_, °C	Δ*H*^PLA^_cc_, J g^−1^	Δ*H*^PLA^_m_, J g^−1^	Δ*H*^PHO^_m_, J g^−1^	*X*_c_, %
PLA neat	66.9	—	90.8	161.2	15.2	18.5	—	19.8
75PLA-25PHO	66.5	52.9	90.2	160.4	9.4	10.8	3.72	15.4
50PLA-50PHO	65.7	52.9	85.5	160.4	11.2	16.9	2.11	36.1
40PLA-60PHO	66.1	52.9	86.5	161.0	9.7	11.3	5.00	30.3

### 3.5. TG analysis

Thermal degradation of the neat PLA and PLA/PHO fiber blends of different composition, where the weight loss due to the volatilization of degradation products was monitored as a function of temperature, is presented in Fig. 4, while the characteristic temperatures (*T*_onset_ at 10% of weight loss, *T*_50%_ at 50% of weight loss, maximum degradation temperatures *T*_d_1__ and *T*_d_2__, *T*_end_ when the degradation ended) are listed in Table 3. From the Fig. 4, one-step degradation was indicative for the neat PLA fibers with the onset degradation temperature at 302 °C and maximum degradation temperature at 352 °C which was in line with the previously reported study.^28^ As was suggested, PLA degrades in one step due to the intramolecular transesterification reaction that occurs above 300 °C.^29^ Contrary to the one-step degradation of the neat PLA, the PLA/PHO fiber blends showed two-step degradation mechanism. Taking into account that pure PHO shows maximum degradation rate at 280 °C,^30,31^ the first degradation stage is related to PHO degradation while the second stage happened at higher temperatures, and it is attributed to the degradation of PLA. In the DTGA curve of the fibers with the highest PHO content, 40PLA-60PHO, additional peak at approximately 303 °C was detected coming from the 3-hydroxyhexanoate fractions contained in the PHO.^32^ This peak, indicating three-step degradation mechanism of sample, was only observed in the fibers with 60% of PHO. From Fig. 4B, DTG curves showed that both PHO and PLA maximum degradation temperatures (*T*_d_1__ and *T*_d_2__) were affected by the presence of other polymer in the blend fibers. More precisely, the increase in PLA content resulted in the decrease of *T*_d_1__ values (75PLA-25PHO had *T*_d_1__ of 280 °C contrary to 40PLA-60PHO with *T*_d_1__ of 288 °C), while the thermal stability of PLA was improved in the presence of PHO polymer, hence the fiber blend with the highest PHO content showed the *T*_d_2__ of 371.6 °C which was about 10 °C higher in comparison to *T*_d_2__ of 75PLA-25PHO fibers (362.6 °C).

**Table 3:** TGA and DTGA results (characteristic temperatures and residual weights at 600 °C, *w*)

Sample	*T*_onset_, °C	*T*_50%_, °C	*T*_end_, °C	*T*_d_1__, °C	*T*_d_2__, °C	*w*, %
PLA	302.2	339.9	362.9	—	352.0	1.60
75PLA-25PHO	271.9	327.8	378.9	280.3	362.6	1.55
50PLA-50PHO	274.3	309.6	381.4	284.8	365.6	1.20
40PLA-60PHO	281.6	338.7	385.2	288.6/302.9	371.6	3.40

**Table 4:** Results of the mechanical tests of PLA and PLA/PHO fibers and average pore size and porosity

Sample	Young's modulus, MPa	Stress at break, MPa	Elongation at break, %	Average pore size ± SD, μm^2^	Porosity (%)
PLA	0.16 ± 0.01	0.20 ± 0.01	8.96 ± 0.21	12.9 ± 4.01	40.6
40PLA-60PHO	2.48 ± 0.11	1.63 ± 0.15	6.05 ± 0.15	7.82 ± 3.07	17.9
50PLA-50PHO	2.36 ± 0.65	1.52 ± 0.35	3.23 ± 0.13	3.24 ± 1.21	19.2
75PLA-25PHO	3.02 ± 0.01	2.06 ± 0.08	4.82 ± 0.04	5.70 ± 2.01	50.9

### 3.6. XRD analysis

**Fig. 5:**
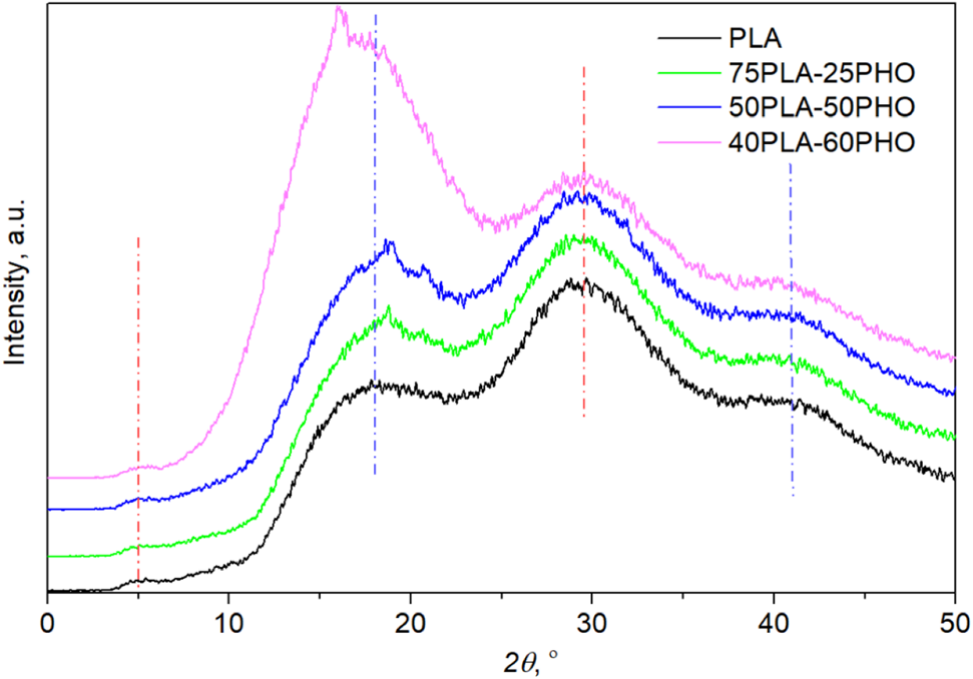
XRD diffractograms of the neat PLA and PLA/PHO fibers with increasing PHO content.

XRD patterns (Fig. 5) confirmed the amorphous state for all materials after processing since no diffraction peaks but only the amorphous halo was observed. The crystallinity patterns of the prepared neat PLA and PLA/PHO fibers were estimated by XRD analysis. Based on the diffraction patterns presented in Fig. 5, the absence of any sharp, diffraction peaks could be noticed confirming the amorphous structure of fibers. Neat PLA indicated three typical broad peaks at *2θ* angles of 17.9°, 29.6° and 41.1° indicating amorphous structure previously reported for PLA based fibers.^33^ Small intensity, broad peak at around 5° was also visible in the diffractograms of all samples. When PLA is in crystalline form, the characteristic diffraction peaks appeared at *2θ* angles near 17° ascribed to α crystals, near 24° associated with β phase of characteristic orthorhombic crystal lattice.^34^ After blending with PHO, no shifting of PLA characteristic peaks was detected, confirming that these materials maintained their amorphous structure. However, some additional peaks appeared around *2θ* of 16.1° and 18.8° that could both be attributed to the presence of PHO in fibers^35^ but also presence of PHO might induce better crystallization and higher ordered polymer chains organization in PLA/PHO fibers which is consistent with the obtained DSC degrees of crystallinity. In the case of fiber with the highest amount of PLA, 75PLA-25PHO, only small, sharp peak at 18.8° is detected and the appearance of diffractogram was quite like those recorded for neat PLA fibers. Higher amount of PHO polymer in blends meant narrower amorphous peak, whereas for the sample with the highest PHO content, additional sharp peak appeared at *2θ* of 16.1°. Despite the small diffraction peaks in PLA/PHO fibers indicative of samples crystallinity, presented XRD patterns were preferentially amorphous, broad peaks hence the degree of crystallinity was difficult to calculate from the obtained XRD results.

### 3.7. Mechanical analysis

Mechanical properties were investigated in terms of tensile strength, Young's modulus and elasticity, and the stress/strain curves were presented in Fig. S2,† while in Table 4, mechanical tests results were summarized. Young's modulus, indicating the stiffness or rigidity of the material, increased in PLA/PHO fibers compared to neat PLA, with the highest value calculated for the 75PLA-25PHO fiber, while the other two polymer blend PLA/PHO fibers had quite similar Young's modulus values. In general, all the obtained elastic modulus were quite low in comparison to similar PLA fiber based studies^36^ which is characteristic property for rubber-like amorphous polymers but also is expected for the fiber-like membranes which indicated lower elasticity compared to casted membranes obtained from the same polymer.^37^ Therefore, those results were consistent with DSC and XRD analysis, where low crystallinity and amorphous structure were confirmed. The highest capacity to resist breaking, stress at break, showed 75PLA-25PHO (2.06 MPa), with the highest PLA content, while this value was remarkably lower for the neat PLA (0.20 MPa). Contrary to the trend noticed for elasticity modulus and stress at break values, when the higher amount of PLA in polymer fiber blends resulted in the increase of these values, elongation at break was the highest for neat PLA fibers. It is important to emphasize that small splitting in the stress/strain curve of neat PLA is due to non-simultaneous breaking of individual fibers in the structure of the sample. In the series of PLA/PHO fiber mats, the elongation at break of 40PLA-60PHO was significantly higher compared to other two PLA/PHO fibers, pointing to an important enhancement in ductility by blending PLA and PHO polymers. This finding was not surprising knowing that material ductility is strongly correlated with *T*_g_ value – higher glassing temperature resulted in the lower elongation at break value^37^ hence those results are in agreement with those obtained from DSC analysis.

### 3.8. Cellular studies

Cellular studies are crucial for materials with medical applications for numerous reasons. Materials used in medical applications come into direct contact with cells and tissues in the human body. Therefore, it is essential to study how the material interacts with these cells and tissues to ensure its safety and efficacy.^11^ Widely performed cellular studies can provide insight into the material's performance and functionality. Studying the interaction between a biomaterial and cells, it can be determined how well the material promotes tissue regeneration and wound healing. *In vitro* cellular experiments are crucial for materials with medical applications and can provide valuable information that can help improve the design and development of final product.

#### 3.8.1. Cytotoxicity

**Fig. 6:**
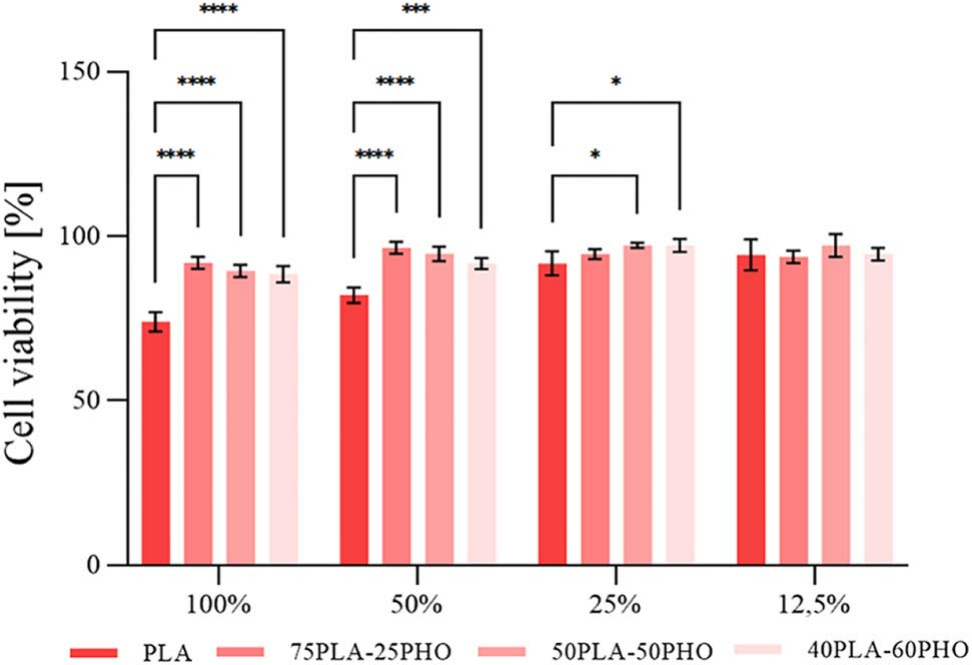
Cell viability as a function of biomaterial formulation and extract concentration (%, v/v); significant differences compared to the control, the neat PLA fiber sample, for the same dilution are indicated by asterisk (*p ≤ 0.05, **p ≤ 0.01, ***p ≤ 0.001, ****p ≤ 0.0001).

Cytotoxicity evaluation was performed by MTT assay using MRC5 cells (human lung fibroblasts obtained from ATCC) and filtered biomaterial extract. Results are presented in Fig. 6. The addition of PHO has improved biocompatibility of the obtained polymer blend biomaterials, because there is a statistically significant difference in cell viability upon the treatment with PLA *vs.* PLA/PHO samples. This effect was irrespective of the PHO content. Similar finding of biocompatibility enhancement of fibrous matrices upon addition of more biocompatible constituent like collagen was also reported for coaxial polyurethane/collagen compound nanofibers.^38^

#### 3.8.2. Cellular morphology

**Fig. 7:**
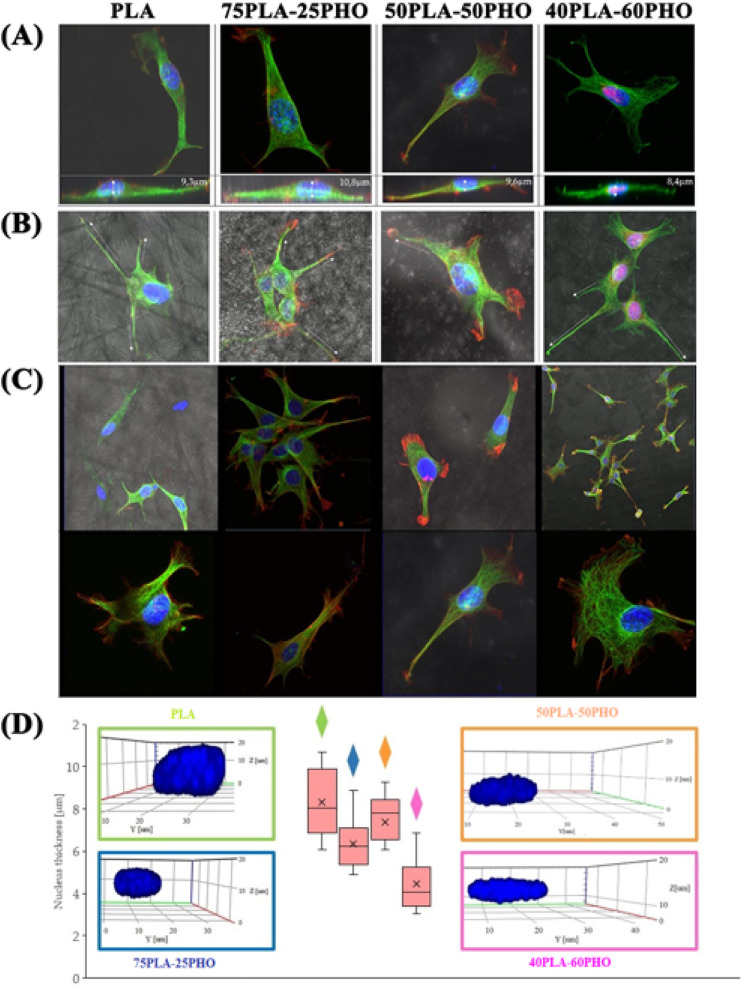
Confocal microscopy imaging: a 3D reconstruction of the cells incubated on different formulations of the PLA/PHO biomaterials: (A) top and side view of the cells; (B) filopodia extension along the biomaterial fibers; (C) cytoskeleton: microtubules – green, actin filaments – red; and (D) cell nucleus thickness as a function of the PHO content in the fibers. 3D reconstructed nuclei are marked in blue.

Cellular morphology studies have supported these findings as well. The influence of the material on cell cytoskeleton morphology was investigated by confocal microscopy. Scaffold properties significantly affect behavior and morphology of the seeded cells, especially in the case of materials with complex architecture where cells undergo numerous interactions mediated by adhesive structures.^39^ Flattening of the cells and filopodia formation was observed on all sample formulations, which is in accordance with the literature data regarding cell incubation on the same polymers.^40^ However, this effect was more pronounced on the PLA/PHO fibers, and it was proportional to the PHO content in the sample (Fig. 7a–c). The higher the PHO content, the better the adhesion of the cells onto the substrates. The positive influence of the PHO on cell adhesion and spreading was most obvious after 3D reconstruction of the cell nuclei. A gradual change in the nuclei shape (from round to flattened) and the corresponding thickness decrease were observed as the PHO content in the polymer blend fibers increased from 0 to 60 wt% (Fig. 7d). In contrast to this, morphology of the cells incubated on the neat, solvent cast PHO films was not significantly changed.^8^ Despite good adherence and spreading, they retained round nuclei and height meaning that the scaffold architecture is an important aspect of the biomaterial biocompatibility.

Changes in cellular morphology and locomotion are mediated by changes in the cytoskeleton. For all biomaterial formulations, the microtubular network was well defined throughout the cell volume and the thickest filaments were located on the cell-substrate attachment points (Fig. 7a–c). The increase in the PHO content in the fibers has led to the changes in morphology and organization of the microtubules – they had smaller diameter and were more densely packed. Well-defined filaments of this cytoskeleton component were also observed in the lower and upper parts of the cell nucleus (Fig. 7c).

**Fig. 8:**
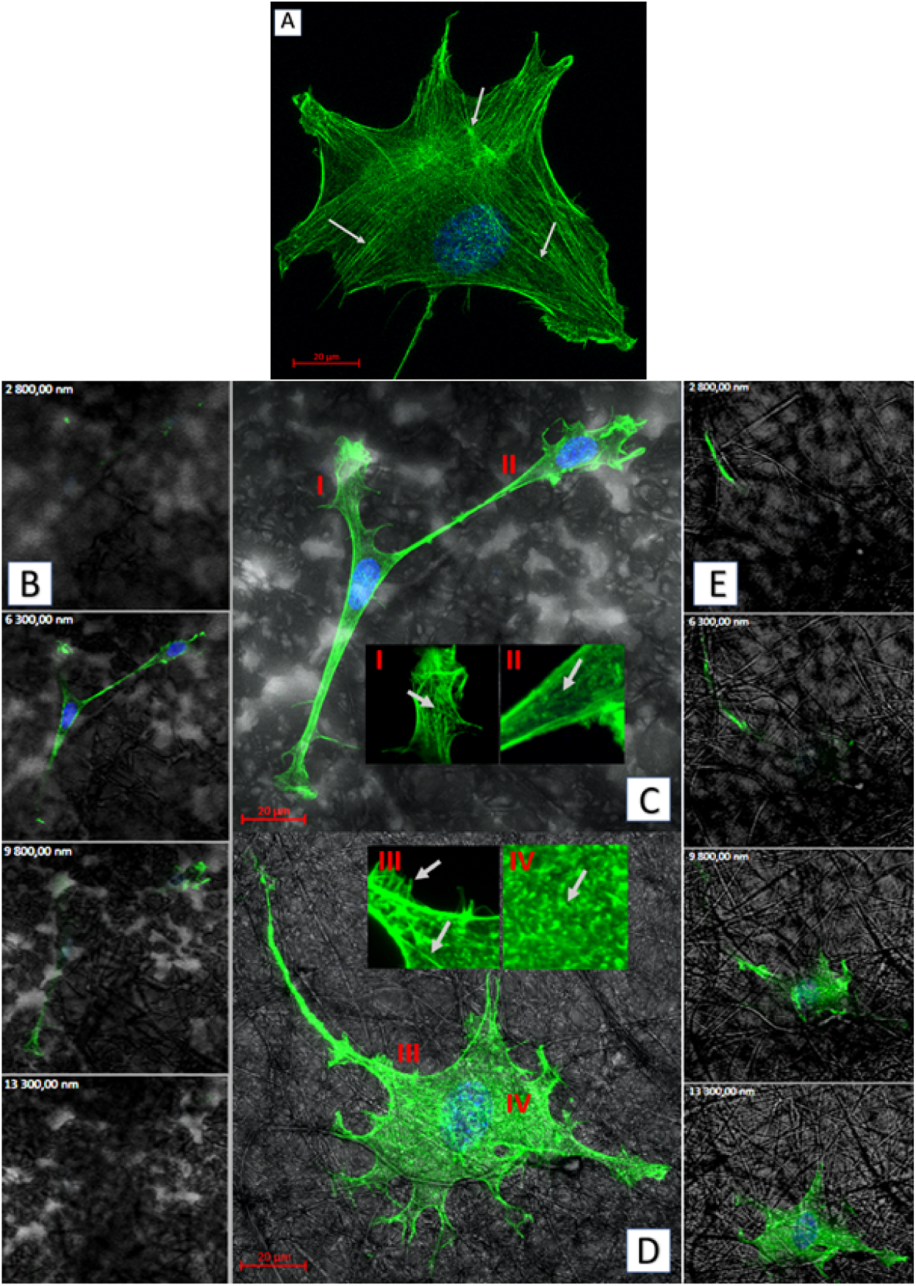
Exemplary images obtained by fluorescent confocal microscopy (actin cytoskeleton-green, nuclei-blue, biopolymer substrate structure-gray): (A) top view of the fibroblast cultured on the glass; (B) sequence of successive focal planes of cells incubated on the 40PLA-60PHO blend; (C) top view of the cells cultured on the 40PLA-60PHO, with the magnifications of the chosen cytoskeleton structures (I and II); (D) top view of the cell cultured on the 75PLA-25PHO, with the magnifications of the chosen cytoskeleton structures (III and IV); (E) sequence of successive focal planes of cell incubated on the 75PLA-25PHO blend.

As for the actin which is the cytoskeleton component responsible for the shape of the cell and its ability to migrate,^8^ significant changes in its structure were observed, depending on the applied culturing substrate. Confocal microscopy was used to visualize the network of actin filaments and cell cultures on glass substrate served as a reference. Fibroblast cells grown in standard conditions (*i.e.* on a glass substrate) frequently show a spindle-shaped morphology with a clearly marked leading edge, or strongly flattened morphology with visible protrusions spread on each side.^40^ Distinct, long actin filaments are located throughout the cell. Rear of the cell body with ventral stress fibers and the front of it including radial and transversal actin filaments are clearly visible. A small amount of shorter actin chains occurs between them, distinguishable as granular structures (Fig. 8a). Morphology of the actin cytoskeleton of fibroblasts grown on the PLA/PHO blends is significantly different from the one presented as the reference sample. The cells grown on the 40PLA-60PHO material were only characterized by spindle-shape, expanded shape and a long trailing edge. Well-flattened and adhered protrusions, equipped with a well-organized, dense network of thin actin filaments were observed, unlike in the rest of the cell body, where the microfilaments mesh is much less defined and most of the volume is occupied by shorter actin chains. In cultures seeded on 40PLA-60PHO biomaterials, typical stress fibers found in cells on glass were not defined (Fig. 8c I and II). In the case of fibroblasts grown on a medium with the higher PLA content: 75PLA-25PHO, the shape of the actin cytoskeleton differed significantly both from those grown on the 40PLA-60PHO blend and on the glass. In the sample containing this biopolymer substrate, the cells represented highly differentiated morphologies. The influence of the substrate fibers on the arrangement of fibroblasts actin cytoskeleton is clearly visible in majority of the cells, as well as a significant increase in the frequency of thin protrusions, with many bifurcations, often running along or next to the filaments of the biopolymer blend. At the same time, the presence of very long trailing edge-like structures was also observed (Fig. 8d). When it comes to the structure of the microfilament network itself inside the cells, no distinct stress fibers were distinguished in the case of the substrate with the higher PLA content. Few thin filaments, predominantly stretched along the length of the substrate filaments, were found, however numerous disorganized short actin chains throughout the cell body were observed (Fig. 8d III and IV). Similar findings were reported for the film substrates made from PLA and PHO^40,41^ and for fiber-reinforced hydrogel composites.^42^ All materials have no negative effect on mammalian cells. In the case of materials made of pure PLA and containing 75% of this polymer in their composition, the cells penetrated the structure of the material less and showed lower tendency to flatten. These features would indicate these materials for use as a dressing, additionally enriched, for example, with a controlled drug release system. Materials with a higher PHO content will be better suited for tissue scaffolds that can be integrated into the regenerated tissue. The material with the same content of PLA and PHO showed intermediate properties, like the material with 60% PLA content, it is most useful for medical applications. The cells behavior on this substrate does not clearly indicate whether it would be better for dressings or scaffolds.

**Fig. 9:**
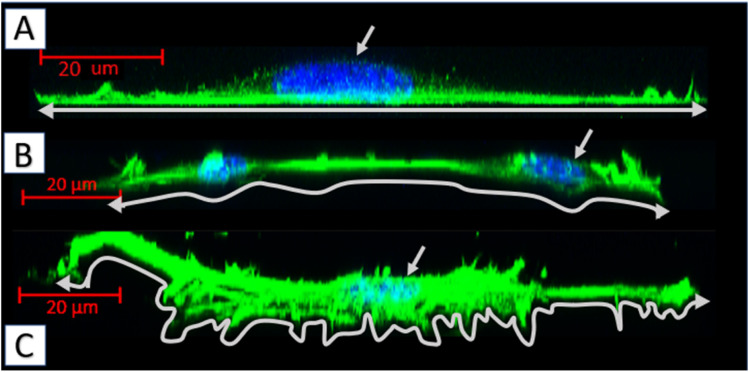
Side projections of the MEF 3T3 cells, obtained by fluorescent confocal microscopy (actin cytoskeleton-green, nuclei-blue): (A) cell cultured on the glass; (B) cells cultured on the 75PLA-25PHO blend; (C) cell cultured on the 40PLA-60PHO blend.

Biopolymer substrates made by electrospinning have undoubtedly a multidimensional structure, which has a key impact on the behavior of cells.^43,44^ A significant tendency of mouse embryonic fibroblasts to migrate not only on the surface of biopolymer substrates, but also in their depths, was noted. During the microscopic measurements, the presence of cells was detected at different depths (focal planes) of the tested specimens. The tendency to invade the multidimensional substrate could also be observed using single cells. Thanks to the confocal microscopy, it was possible to observe that the cells were located among the fibers of the substrate with numerous protrusions reaching deep into the material (Fig. 8b and e). This was also confirmed by the side projections of the cells seeded on different substrates (Fig. 9).

The fibroblasts cultured on glass adhered homogenously to it, without any protrusions (Fig. 9a). On multidimensional biopolymer substrates, as previously shown, cells have exhibited slightly different morphologies. On the substrate made of 75PLA-25PHO, the cells adhered closely to the culture material, adapting the shape of the microfilament network to its structure. A few, thin protrusions penetrating the porous structure of the substrate were observed (Fig. 9b). When cells were grown on the 40PLA-60PHO biomaterial, numerous protrusions with a built-in actin cytoskeleton were observed. These protrusions were directed both in the direction parallel to the substrate, along the biopolymer filaments-filopodia and lamellipodia, and deep into its structure, forming the so-called invadopodia.^45^ In addition, numerous cells have shown a polarized shape suggesting the direction of migration, as well as a clearly shaped retraction tail that was located above or below the cell placed in the network of substrate fibers. This observation may prove that in the case of a substrate with a higher content of PHO, the cells not only penetrate the material with the help of invadopodium, but also migrate throughout the biopolymer fibers (Fig. 9c). Furthermore, significant differences in the actin cytoskeleton architecture were observed in the cell nucleus area. In the cells on glass, the microfilaments above the nucleus were negligible, while in the case of fibroblasts grown on the PLA/PHO substrates, the actin network clearly covers the surface of the nucleus. The thickest layer of actin enveloping cell nuclei was observed in the case of a cells grown on medium made of 75PLA-25PHO blend (Fig. 9a–c). Similar differences were reported in literature before.^8,11^

#### 3.8.3. Cell migration

**Fig. 10:**
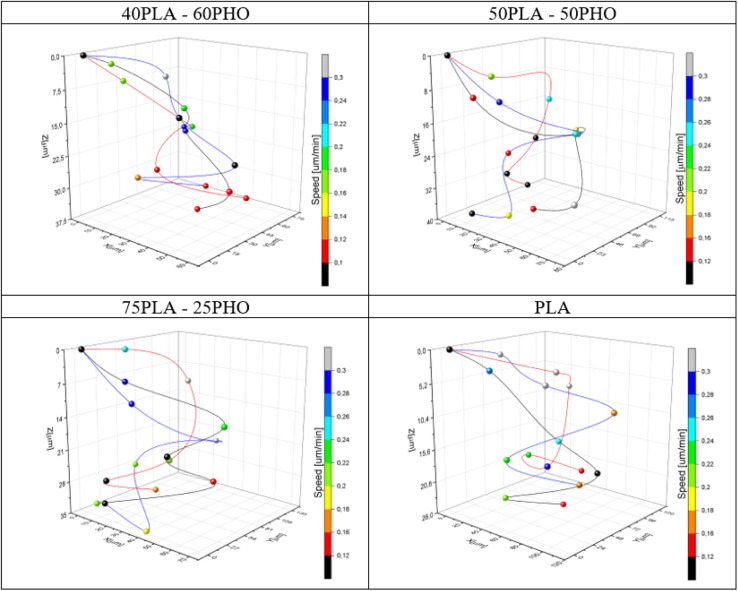
Exemplary cell movement 3D trajectories recorded during 24 hours of observation for each material. The instantaneous speed of the cells is coded with color of the markers.

Cell migration is a complex process that involves the movement of cells within tissues or through extracellular matrix.^46^ It is essential for various physiological processes, such as embryonic development, wound healing, and immune responses. Cells migrate by using various mechanisms, including actin-driven protrusions, cell-substrate adhesion, and cytoskeletal rearrangements.^8^ The process of cell migration plays a crucial role in wound healing. During this process, cells migrate into the wound site and proliferate to promote tissue repair. One promising approach is the use of biomaterials that mimic the extracellular matrix to promote cell migration. Biomaterials such as PLA and PHO play a critical role in the development of wound dressings due to their unique properties, including biocompatibility, biodegradability, and the ability to support cell growth and tissue regeneration.^11,47^ It is therefore important to understand the effect of tested materials on cellular migration. In this work, it was decided to use a three-dimensional analysis that allows, in addition to the movement of cells on the surface, to illustrate the penetration of the biomaterial internal structure, as this process is crucial for the assumed integration of the dressing with the surrounding tissues. Exemplary cell movement 3D trajectories recorded during 24 hours of observation are presented for each material in Fig. 10.

**Fig. 11:**
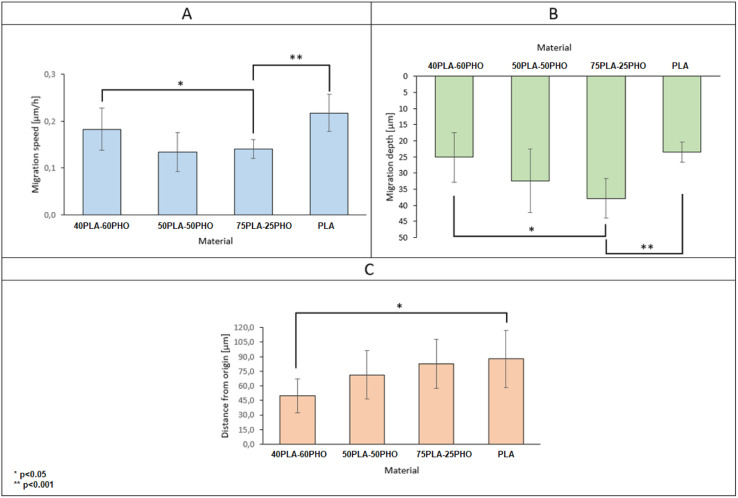
Basic parameters of cell migration on electrospun materials with different formulations.

By collecting complete data cell migration, a detailed analysis was possible showing the influence of the medium composition on cell migration. It should be noted that the analysis in the “*Z*” axis (depth of penetration), which is particularly important for the materials considered in this study, was also performed. Due to their biodegradability and biocompatibility, dressing materials made of the studied polymers can be, potentially, used as a scaffold that can be infiltrated by cells in wound, supporting in turn regeneration and undergo a natural decomposition following the wound healing. Quantitative results for migration are presented in Fig. 11. For each material, 20 cells were selected, whose parameters were recorded within 24 hours and then averaged and compared between materials.

**Fig. 12:**
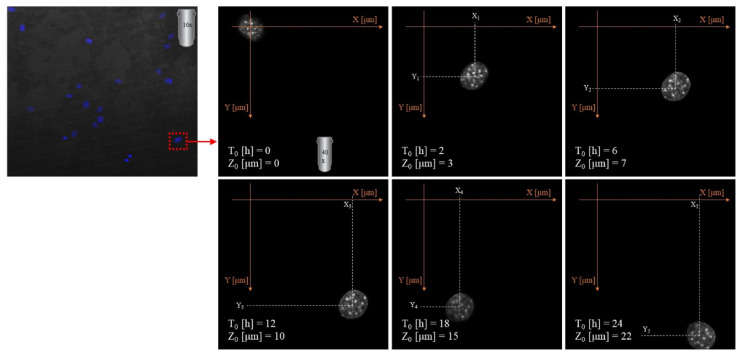
Exemplary cell migration pathway analysis presented on microscopic images obtained during the measurements.

The analysis of migration parameters has shown that a greater content of PLA causes the acceleration of cell migration (Fig. 11A). This is consistent with the results of mechanical analysis (Table 4) and the previous observations correlating the Young's modulus of the substrate with the migration velocity.^41,48,49^ The opposite effect can be observed in the case of migration depth analysis. Cells cultured on the fibers richer in PHO tend to penetrate deeper into the 3D structure of the material. In the case of pure polylactide, the maximum average depth at which cells were found after 24 hours is 23.5 μm, while for the substrate containing 60% PHO is 37.9 μm (Fig. 11B). It was also found that cells seeded on harder substrates containing more PLA tend to move more directional, which causes their final position to be further from the starting point (Fig. 11C). This effect may be due to the fact that the cells treat polyhydroxyalkanoate substrates as nutrient,^21,38^ therefore their movement is directed to the search for elements built of PHO in the structure of the material. Exemplary time lapse images allowing the quantitative 3D migration analysis are presented (Fig. 12). When it comes to migration and colonization of the material by cells, the best material for absorbable dressings seems to be the one with the highest PHO content. The addition of PLA causes a slight increase in Young's modulus, which in turn accelerates the rate of migration and thus the healing of the wound. Composites of these two materials in a 40 : 60 (PLA/PHO) ratio seem to be an ideal solution for further research.

## 4. Conclusions

PHO was successfully electrospun into biomimetic fibrous scaffolds by blending with PLA. The presence of elastomeric, biocompatible constituent – PHO improves properties and overall biocompatibility of the obtained biomaterials, exceptionally supports adhesion and migration of fibroblasts which is a prerequisite for cellular migration and synthetic activity. This methodological approach paved the way for mcl-PHAs to be increasingly utilized for this high-end application, as obtained biomaterials can be used as biocompatible, biodegradable, and sustainable biomaterials for drug delivery or tissue engineering. This work clearly indicates that 3D materials that can offer precise control over the shape, size, and porosity of implants, will be the future of medical devices allowing for personalized and optimized medical treatments. Such materials can be tailored to mimic the natural properties of tissues and organs, which can aid in tissue engineering and regenerative medicine. Thanks to use of biocompatible, biodegradable materials patients' recovery may be improved, and inflammation risk minimized. Application of advanced 3D materials can enable the creation of complex medical devices and instruments with intricate geometries that are not possible with traditional manufacturing techniques.

## Conflicts of interest

There are no conflicts to declare.

## Supplementary Material

RA-013-D3RA03021K-s001

## References

[cit1] Zinn M., Witholt B., Egli T. (2001). Occurrence, synthesis and medical application of bacterial polyhydroxyalkanoate. Adv. Drug Delivery Rev..

[cit2] Nikodinovic-Runic J. , Guzik M., Kenny S. T., Babu R., Werker A., and O'Connor K. E., Carbon-rich wastes as feedstocks for biodegradable polymer (polyhydroxyalkanoate) production using bacteria, in Advances in Applied Microbiology, Academic Press Inc., 2013, pp. 139–200, 10.1016/B978-0-12-407673-0.00004-7 23763760

[cit3] Anjum A., Zuber M., Zia K. M., Noreen A., Anjum M. N., Tabasum S. (2016). Microbial production of polyhydroxyalkanoates (PHAs) and its copolymers: a review of recent advancements. Int. J. Biol. Macromol..

[cit4] Savenkova L., Gercberga Z., Bibers I., Kalnin M. (2000). Effect of 3-hydroxy valerate content on some physical and mechanical properties of polyhydroxyalkanoates produced by Azotobacter chroococcum. Process Biochem..

[cit5] Radivojevic J. (2016). *et al.*
, Polyhydroxyalkanoate-based 3-hydroxyoctanoic acid and its derivatives as a platform of bioactive compounds. Appl. Microbiol. Biotechnol..

[cit6] Li W., Cicek N., Levin D. B., Liu S. (2019). Enabling electrospinning of medium-chain length polyhydroxyalkanoates (PHAs) by blending with short-chain length PHAs. Int. J. Polym. Mater. Polym. Biomater..

[cit7] Maleki H., Azimi B., Ismaeilimoghadam S., Danti S. (2022). Poly(lactic acid)-Based Electrospun Fibrous Structures for Biomedical Applications. Appl. Sci..

[cit8] Witko T., Solarz D., Feliksiak K., Rajfur Z., Guzik M. (2019). Cellular architecture and migration behavior of fibroblast cells on polyhydroxyoctanoate (PHO): a natural polymer of bacterial origin. Biopolymers.

[cit9] Wan Y., Chen W., Yang J., Bei J., Wang S. (2003). Biodegradable poly(L-lactide)-poly(ethylene glycol) multiblock copolymer: Synthesis and evaluation of cell affinity. Biomaterials.

[cit10] Davachi S. M., Kaffashi B. (2015). Polylactic Acid in Medicine. Polym.-Plast. Technol. Eng..

[cit11] Witko T., Solarz D., Feliksiak K., Haraźna K., Rajfur Z., Guzik M. (2020). Insights into in vitro wound closure on two biopolyesters –polylactide and polyhydroxyoctanoate. Materials.

[cit12] Vu L. T., Jain G., Veres B. D., Rajagopalan P. (2015). Cell Migration on Planar and Three-Dimensional Matrices: A Hydrogel-Based Perspective. Tissue Eng., Part B.

[cit13] Sofińska K. (2019). *et al.*
, Structural, topographical, and mechanical characteristics of purified polyhydroxyoctanoate polymer. J. Appl. Polym. Sci..

[cit14] Schindelin J. (2012). *et al.*
, Fiji: an open-source platform for biological-image analysis. Nat. Methods.

[cit15] Zimmerling A., Yazdanpanah Z., Cooper D. M. L., Johnston J. D., Chen X. (2021). 3D printing PCL/nHA bone scaffolds: exploring the influence of material synthesis techniques. Biomater. Res..

[cit16] Ferreira T. and Rasband W., ImageJ User Guide, 2012

[cit17] Katzenberg F., Tiller J. C. (2016). Shape memory natural rubber. J. Polym. Sci., Part B: Polym. Phys..

[cit18] Jaiswal M., Koul V. (2013). Assessment of multicomponent hydrogel scaffolds
of poly(acrylic acid-2-hydroxy ethyl methacrylate)/gelatin for tissue engineering applications. J. Biomater. Appl..

[cit19] Tamm C., Galitó S. P., Annerén C. (2013). A comparative study of protocols for mouse embryonic stem cell culturing. PLoS One.

[cit20] Koski A., Yim K., Shivkumar S. (2004). Effect of molecular weight on fibrous PVA produced by electrospinning. Mater. Lett..

[cit21] Xue J., Wu T., Dai Y., Xia Y. (2019). Electrospinning and electrospun nanofibers: Methods, materials, and applications. Chem. Rev..

[cit22] Natarajan L., New J., Dasari A., Yu S., Manan M. A. (2014). Surface morphology of electrospun PLA fibers: Mechanisms of pore formation. RSC Adv..

[cit23] Malagurski I. (2021). *et al.*
, Polyhydroxyoctanoate films reinforced with titanium dioxide microfibers for biomedical application. Mater. Lett..

[cit24] Oztemur J., Yalcin-Enis I. (2021). Development of biodegradable webs of PLA/PCL blends prepared via electrospinning: Morphological, chemical, and thermal characterization. J. Biomed. Mater. Res., Part B.

[cit25] Wu D., Samanta A., Srivastava R. K., Hakkarainen M. (2018). Nano-graphene oxide functionalized bioactive poly(lactic acid) and poly(ε-caprolactone) nanofibrous scaffolds. Materials.

[cit26] Bagdadi A. V. (2018). *et al.*
, Poly(3-hydroxyoctanoate), a promising new material for cardiac tissue engineering. J. Tissue Eng. Regener. Med..

[cit27] Alhaddad O., El-Taweel S. H., Elbahloul Y. (2019). Nonisothermal Cold Crystallization Kinetics of Poly(lactic acid)/Bacterial Poly(hydroxyoctanoate) (PHO)/Talc. Open Chem..

[cit28] Alhaddad O., El-Taweel S. H., Elbahloul Y. (2019). Nonisothermal Cold Crystallization Kinetics of Poly(lactic acid)/Bacterial Poly(hydroxyoctanoate) (PHO)/Talc. Open Chem..

[cit29] Muthuraj R., Misra M., Mohanty A. K. (2018). Biodegradable compatibilized polymer blends for packaging applications: A literature review. J. Appl. Polym. Sci..

[cit30] Alhaddad O., El-Taweel S. H., Elbahloul Y. (2019). Nonisothermal Cold Crystallization Kinetics of Poly(lactic acid)/Bacterial Poly(hydroxyoctanoate) (PHO)/Talc. Open Chem..

[cit31] Liu X., Gao C., Sangwan P., Yu L., Tong Z. (2014). Accelerating the degradation of polyolefins through additives and blending. J. Appl. Polym. Sci..

[cit32] Li D., Fu J., Ma X. (2020). Improvement in thermal, mechanical, and barrier properties of biocomposite of poly (3-hydroxybutyrate-co-3-hydroxyhexanoate)/modified nano-SiO 2. Polym. Compos..

[cit33] Puchalski M., Kwolek S., Szparaga G., Chrzanowski M., Krucinska I. (2017). Investigation of the influence of PLA molecular structure on the crystalline forms (α’’ and α) and Mechanical Properties ofWet Spinning Fibres. Polymers.

[cit34] Oliveira J. E., Mattoso L. H. C., Orts W. J., Medeiros E. S. (2013). Structural and Morphological Characterization of Micro and Nanofibers Produced by Electrospinning and Solution Blow Spinning: A Comparative Study. Adv. Mater. Sci. Eng..

[cit35] Li W., Cicek N., Levin D. B., Liu S. (2019). Enabling electrospinning of medium-chain length polyhydroxyalkanoates (PHAs) by blending with short-chain length PHAs. Int. J. Polym. Mater. Polym. Biomater..

[cit36] Li W., Cicek N., Levin D. B., Liu S. (2019). Enabling electrospinning of medium-chain length polyhydroxyalkanoates (PHAs) by blending with short-chain length PHAs. Int. J. Polym. Mater. Polym. Biomater..

[cit37] Armentano I. (2015). *et al.*
, Processing and characterization of plasticized PLA/PHB blends for biodegradable multiphase systems. eXPRESS Polym. Lett..

[cit38] Chen R., Huang C., Ke Q., He C., Wang H., Mo X. (2010). Preparation and characterization of coaxial electrospun thermoplastic polyurethane/collagen compound nanofibers for tissue engineering applications. Colloids Surf., B.

[cit39] Do A.-V., Khorsand B., Geary S. M., Salem A. K. (2015). 3D Printing of Scaffolds for Tissue Regeneration Applications. Adv. Healthcare Mater..

[cit40] Witko T., Solarz D., Feliksiak K., Haraźna K., Rajfur Z., Guzik M. (2020). Insights into In Vitro Wound Closure on Two Biopolyesters—Polylactide and Polyhydroxyoctanoate. Materials.

[cit41] Feliksiak K., Solarz D., Guzik M., Zima A., Rajfur Z., Witko T. (2021). Vimentin Cytoskeleton Architecture Analysis on Polylactide and Polyhydroxyoctanoate Substrates for Cell Culturing. Int. J. Mol. Sci..

[cit42] Matera D. L., Wang W. Y., Smith M. R., Shikanov A., Baker B. M. (2019). Fiber Density Modulates Cell Spreading in 3D Interstitial Matrix Mimetics. ACS Biomater. Sci. Eng..

[cit43] Ochsner M., Textor M., Vogel V., Smith M. L. (2010). Dimensionality Controls Cytoskeleton Assembly and Metabolism of Fibroblast Cells in Response to Rigidity and Shape. PLoS One.

[cit44] Doyle A. D., Petrie R. J., Kutys M. L., Yamada K. M. (2013). Dimensions in cell migration. Curr. Opin. Cell Biol..

[cit45] Jacquemet G., Hamidi H., Ivaska J. (2015). ScienceDirect Filopodia in cell adhesion , 3D migration and cancer cell invasion. Curr. Opin. Cell Biol..

[cit46] Kraning-Rush C. M., Carey S. P., Califano J. P., Smith B. N., Reinhart-King C. A. (2011). The role of the cytoskeleton in cellular force generation in 2D and 3D environments. Phys. Biol..

[cit47] Sofińska K. (2019). *et al.*
, Structural, topographical, and mechanical characteristics of purified polyhydroxyoctanoate polymer. J. Appl. Polym. Sci..

[cit48] Solon J., Levental I., Sengupta K., Georges P. C., Janmey P. A. (2007). Fibroblast adaptation and stiffness matching to soft elastic substrates. Biophys. J..

[cit49] Hopp I. (2013). *et al.*
, The influence of substrate stiffness gradients on primary human dermal fibroblasts. Biomaterials.

