# Supplementary material for: Biological and physiochemical studies of electrospun polylactid/polyhydroxyoctanoate PLA/P(3HO) scaffolds for tissue engineering applications
Source: RSC Adv. 2023 Aug 11;13(34):24112–28. doi: 10.1039/d3ra03021k (PMC10415749; doi:10.1039/d3ra03021k)
Supplement: RA-013-D3RA03021K-s001 [file RA-013-D3RA03021K-s001.pdf]

Electronic Supplementary Material (ESI) for RSC Advances.
This journal is © The Royal Society of Chemistry 2023



# Supplementary file

**Biological and physiochemical studies of electrospun polylactid/polyhydroxyoctanoate PLA/P(3HO) scaffolds for tissue engineering applications**

Daria Solarz[b], Tomasz Witko[a,c], Robert Karcz[a] Ivana Malagurski[d,*], Marijana Ponjavic[d], Steva Levic[e], Aleksandra Nesic[f], Maciej Guzik[a], Sanja Savic[g] and Jasmina Nikodinovic-Runic[d,*]

[a]*Jerzy Haber Institute of Catalysis and Surface Chemistry Polish Academy of Sciences, Niezapominajek 8, 30-239 Krakow, Poland*

[b]*Faculty of Physics, Astronomy and Applied Computer Science, Jagiellonian University, Lojasiewicza 11, 30-348 Krakow, Poland*

[c]*Department of Product Technology and Ecology, Krakow University of Economics, Rakowicka 27, 31-510 Kraków, Poland*

[d]*Institute of Molecular Genetics and Genetic Engineering, University of Belgrade, 11042 Belgrade, Serbia*

[e]*Faculty of Agriculture, University of Belgrade, 11081 Belgrade, Serbia*

[f]*Faculty of Technology, 21000 Novi Sad, Serbia*

[g]*University of Belgrade, Institute of Chemistry, Technology and Metallurgy, Center of Excellence in Environmental Chemistry and Engineering, Njegoseva 12, 11000, Belgrade, Serbia*

Corresponding authors:

Jasmina Nikodinovic-Runic

Tel: +381 11 397 60 34

E-mail: jasmina.nikodinovic@imgge.bg.ac.rs

and

Tomasz Witko

Tel. +48 507196 866

E-mail: tomasz.witko@ikifp.edu.pl

## 1. Materials and methods

### *1.1. Specific surface area*

The specific surface area ($S_{BET}$) of the samples was calculated using the BET formalism, analyzing the results of nitrogen adsorption/desorption at 77 K. Isotherms were measured using a Nova 2000 Quantachrome instrument, and the data was analyzed using the software provided with the instrument. Prior to the measurements, the samples were outgassed at 40 °C for 20 hours. It is worth mentioning that the use of 100-150 mg of materials in our experiments was necessary due to the limitations in the measuring cell size.

## 2. Results

### *2.1. $S_{BET}$ results*

**Table S1.** Measured surface areas of the fibrous samples.

| Sample | SBET [m$^2$/g] |
|---|---|
| **PLA** | 8.1 |
| **75PLA-25PHO** | 2.0 |
| **50PLA-50PHO** | 2.0 |
| **40PLA-60PHO** | 1.9 |

**Table S2.** Comparison of specific surface areas of fibers and composite materials prepared using biodegradable polymers.

| Sample | S BET [m2/g] | Reference |
|---|---|---|
| PLA | 8.1 | This work |
| **75PLA-25PHO** | 2.0 | |
| **50PLA-50PHO** | 2.0 | |
| **40PLA-60PHO** | 1.9 | |
| PLA/$Al_2O_3$/Ag | n.d. | P. Kurtycz et al.[1] |
| $Al_2O_3$/Ag | 181 | |
| Non porous PLA fiber | 1.45 | Yoon et al.[2] |
| Porous PLA fiber | 1.64 | |
| Non porous fiber with glass beads | 2.90 | |
| Porous with glass beads | 4.39 | |

| | | |
|---|---|---|
| PLA/sepiolite | n.d. | Sabzi et al[3] |
| Sepiolite | 313.6 | |
| PLA/NCC | n.d. | |
| NCC | 17 | |
| Scaffold 1/PLA fibers | 0.5096 | Chung et al.[4] |
| Scaffold 2/PLA fibers | 2.3632 | |
| PLA aerogel | 10.12 | Li et al.[5] |
| PLA | 14.57 | Spiridon et al[6] |
| PLA-4.5 | 26.24 | Wang et al.[7] |
| PLA-5 | 31.00 | |
| PLA-5.5 | 22.45 | |
| PLA-6 | 20.88 | |
| PLLA nanofiber membrane | 4.64 | Bai et al.[8] |
| PLLA nanofiber membrane – plasma treated | 22.84 | |
| P(3HB-co-4HB) | 58.53 | Zhijiang et al[9] |
| Photocatalyst CCZ/P(3HB-co-HHx) fiber | 11.43 | Selvin et al.[10] |
| P(3HB-co-4HB)/CA fiber mat 90:10 | 43.63 | Zhijiang et al.[11] |
| P(3HB-co-4HB)/CA fiber mat 80:20 | 52.34 | |
| P(3HB-co-4HB)/CA fiber mat 70:30 | 58.74 | |
| P(3HB-co-4HB)/CA fiber mat 60:40 | 66.52 | |
| PAN fiber mats | 15.0 | Chen et al.[12] |

*2.2. PLA/PHO fibers wettability*

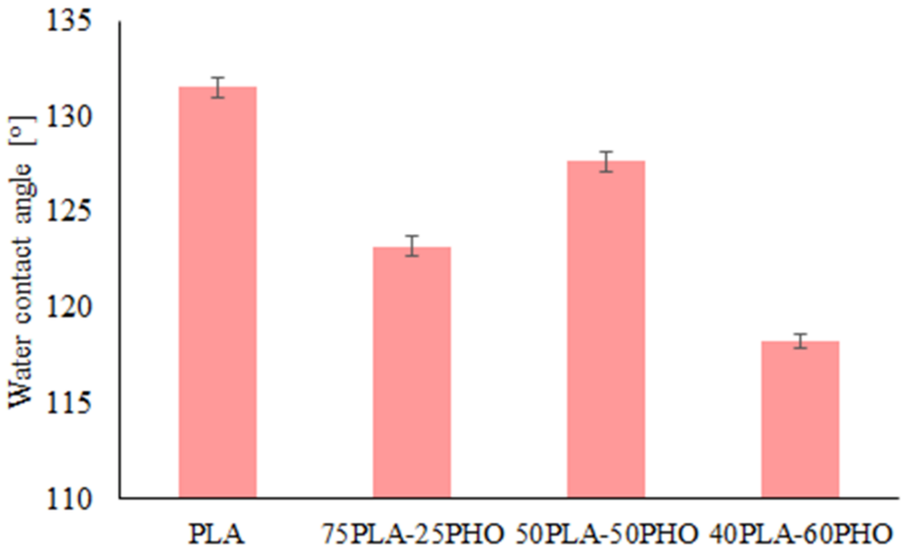


**Figure S1.** Water contact angle of the neat PLA and PLA/PHO fibers with increasing PHO content.

*2.3. Mechanical properties*

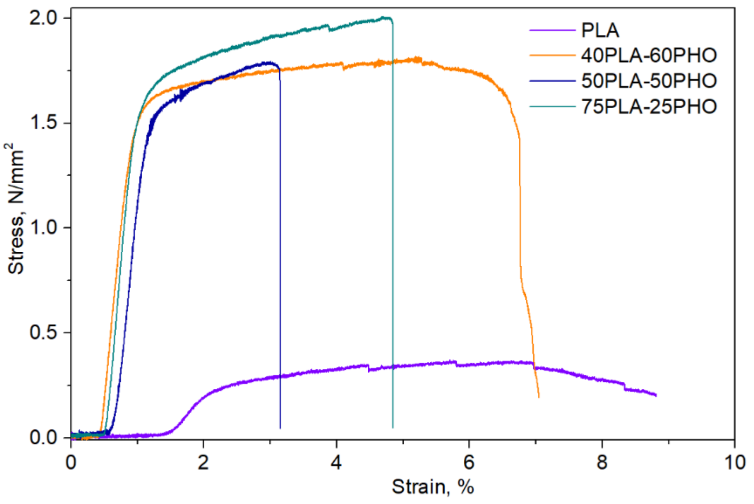


**Figure S2.** Stress/strain curves the neat PLA and PLA/PHO fibers with increasing PHO content.

*2.4. Summary of PHA-based electrospun biomaterials for biomedical application*

Presence of morphology defects is quite frequent phenomenon in PHA-based electrospun materials and is caused, mostly, by changes in electrospinning solution properties upon addition of other constituents (polymers or nanoparticles). Beads were observed in PHB/mcl-PHA[13], terpolymer P(3HB-4HB-3HV)[14], PHBV/PLLA[15], and neat PHBV[16] fibers, while fused regions were reported in P(3HB-*co*-3HH) and P(3HB-*co*-4HB)[17], PHBV/PHOHH[18], PHB/chitosan/bioglass composite[19], and PHBV/olive leaf extract[20] fibers, in the latter case they acted as mini reservoirs of polyphenols. PHBV electrospun fibers obtained using green

solvents derived from levulinic acid were ribbon-like[21]. The addition of PHO has caused a decrease in blend PLA/PHO fiber diameter in our study and similar trend was observed for multiple PHA-based electrospun polymer blends[13], [22]–[24] and composites[19], [25]–[30]. The PLA/PHO blend fibers developed in this study exhibited increase in polymer degree of crystallinity which further reflected on corresponding mechanical and thermal properties. In contrast to this, most of the PHA-derived fibrillar biomaterials exhibited lower degree of crystallinity[13], [15], [22], [27], [29], [31], [32]. However, this was understandable considering that the starting polymer in these instances was either PHB or PHBV, both with much higher degree of crystallinity in comparison to the low crystalline PLA used as a carrier polymer for PHO in this study. In terms of biocompatibility, PLA/PHO fibers proved to be excellent biocompatible biomaterials, which is in agreement with the literature data (**Table S3).**

**Table S3.** Summary of different PHA-based electrospun biomaterials aimed for biomedical application.

| Polymer/blends/composites | Morphology | Thermal properties | Mechanical properties | Degree of crystallinity | WCA | Application | Ref. |
|---|---|---|---|---|---|---|---|
| PHB, PHBV, PHB/PHBV14 % w/v, $CHCl_3$ | Aligned fibers; decrease in diameter (3.7±1.7 to 3.2±1.3 μm). | Decrease in $T_m$ (173.1 to 167.0°C). | Improved upon blending. | Decrease in $\chi_c$ (55.6 to 28.2%). | Increase in WCA (115 to 121.6°). | No cytotoxic effect (mouse fibroblasts L929); biomedical application | Sombatmankhong et al.[22] |
| P(3HB-*co*-3HH), P(3HB-*co*-4HB), 1 wt%, HFIP[a] | Spindle and fused regions; decrease in fiber diameter in comparison to the neat PHB fibers (190 vs. 520 nm). | - | Mechanical properties comparable to human skin. | Copolymer fibers have lower $\chi_c$ (70 and 35% vs. 85% for PHBHH, PHB4HB and PHB fibers, respectively) | - | *In vivo* biodegradable (subcutaneous implantation in rats); skin tissue engineering. | Ying et al.[17] |
| PHBV/AgNP[b], 5 wt%, 2,2,2-trifluoroethyl alcohol | Composite fibers have smaller diameter (630±20 vs. 770±40nm). | - | - | - | - | Antibacterial effect against *Klebsiella pneumoniae* and *Staphylococcus aureus*; supports fibroblasts adhesion and proliferation and osteoblasts differentiation; joint surgery. | Xing et al.[25] |
| PHBV/PDLLA[c], 3 or 6 wt%, $CHCl_3$/DMF[d] | Random and aligned fibers. | A slight increase in $T_m$ (151 to 153°C). | Improved mechanical properties. In random fiber membranes: increased TS 6.0±0.9 to 8.3±0.5MPa, E 0.11±0.01 to 0.13±0.02 Gpa and ε 270±60 to 540±30%. | Decrease in the overall $\chi_c$ (43 to 10%). | - | *In vitro* biodegradable; tunable properties to specific application. | Cheng et al.[31] |
| PHBV/keratin, 6 wt%, HFIP | Decrease in fiber diameter (815±98 to | - | - | - | - | Supports NIH 3 TR cells viability; induces | Yuan et |

| | | | | | | | |
|---|---|---|---|---|---|---|---|
| | 720±124nm). | | | | | wound closure (*in vivo* test in mice); wound dressing/tissue engineering. | al.[33] |
| PHBV/PEO[e], 20 wt%, $CHCl_3$ | Decrease in fiber diameter (2.8±0.1 to 1.3±0.2um); rougher surface due to phase separation. | $T_d$ increase (279 to 285°C) | Decrease in TS, E and ε (1.8±0.2 to 0.4±0.2Mpa, 80±15 to 50±10Mpa, and 30±20 to 10±5%). | Decrease in PHBV $\chi_c$ (53 to 20%). | - | Biomedical application. | Bianco et al.[23] |
| PHB/mcl-PHA, $CHCl_3$/DMF | Decrease in fiber diameter (1.27±0.37 to 0.81±0.37 μm); pronounced beads with mcl-PHA content increase. | - | - | Decrease in $\chi_c$ (47.2 to 28.7%). | - | Biomedical application | Azari et al.[13] |
| Terpolymer P(3HB-4HB-3HV), 4 % w/v, $CHCl_3$ | Beaded morphology; 600-1400 nm average fiber diameter. | - | Mechanical properties in following range: TS: 2.5±0.3 to 11.3±0.9Mpa, E: 73.5±5 to 288±62Mpa, ε: 29±9 to 376±188%. | - | WCA range: 73.1±0.5 to 92.6±0.4°). | No cytotoxic effect (L 929 fibroblast); supports hMSC differentiation into osteogenic and adipogenic phenotype. | Canadas et al.[14] |
| PHB/PLCL[f], 6 wt%, $CHCl_3$/DMF | Decrease in fiber diameter (543.5±145 to 157±96.9 nm). | - | Increase in ε (10.6±1.4 to 55%). | - | Increase in WCA (88.6 to 120°). | Supports adhesion and proliferation of olfactory ensheating cells; neural tissue engineering. | Daranarong et al.[24] |
| PHBV/PLLA, 10 wt%, $CHCl_3$/DMF | Increase in fiber diameter (0.451±0.154 to 1.517±0.368um); beaded morphology with higher PHBV content; porous surface. | A slight decrease in $T_m$ (153 to 148°C); a slight increase in $T_o$ (251.6 to 265.9°C). | Increase in YM (19.9 to. 49.08MPa) and TS (0.35 to 0.78MPa). | Decrease in $\chi_c$ (43.5 to 1.1%). | - | Filtration and biomedical application. | Wagner et al.[15] |
| PHBV/silk fibroin, 10 wt%, HFIP | Blend fibers have smaller diameter (380±65nm to 220-350nm). | - | Increase in TS (1.31±0.2 to 4.84±0.52MPa), YM | - | Decrease in WCA (118.5±1.2 to | Supports mouse fibroblasts L929 adhesion and proliferation. | Lei et al. [26] |

| | | | | | | | |
|---|---|---|---|---|---|---|---|
| | | | (56.5±5.7 to 65.8±4.7MPa) and ε (5.1±0.7 to 44.3±4.0%). | | 70.9±1.9°). | | |
| P(3HB-*co*-4HB)/paclitaxel | Addition of the drug increased the diameter and caused bead free morphology (620±140 to 954±230 nm). | - | - | - | - | Biodegradable *in vitro*; continuous drug release; drug eluting stents. | Sudesh et al.[34] |
| PHBV/PHOHH[g], 15 wt%, $CHCl_3$/DMF | Addition of PHOHH has caused increase in blend fiber diameter (464±94 to 553±115 nm, for 75/25 and 65/35 ratios, respectively) and the appearance of fused regions; optimal ratio 75/25. | - | Decrease in TS (2.90±0.31 to 2.68±0.05 MPa for PHBV and PHBV/PHOHH 75/25, respectively) and YM (41.09±3.69 to 34.46±3.19MPa) and increase in ε (212.186±42.22 to 291.32±51.31 %). | Decrease in $\chi_c$ (20.5 to 16.1%, PHBV vs. PHBV/PHOHH). | - | Mechanical properties match skin, ideal for wound dressings. | Li et al.[18] |
| PHBV, 4 wt%, dichloromethane | Random (1.72±0.52 µm) and aligned (1.56±0.41µm,) fibers; beaded morphology. | - | - | - | - | Tissue engineering scaffolds. | Vega-Castro et al.[16] |
| PHB, PHBV, PHBV/PHOHD[h], 4 wt%, $CHCl_3$, oxygen plasma treatment to improve hydrophilicity | PHB 2.6±0.4 µm; PHBV 2.7±0.4 µm; PHBV/PHOHD 4.0±0.2 µm (70/30); oxygen plasma treatment has decreased the fibers diameter due to physical etching. | Did not change significantly following processing and oxygen plasma treatment. | - | Decrease in $\chi_c$ due to addition of PHOHD (24.7 to 20.9%) and oxygen plasma treatment (38,8; 18,0 and 10.2% for PHB, PHBV and PHBV/mcl fibers, | Initial increase in WCA caused by addition of PHOHD eliminated by oxygen plasma treatment | Supports human dermal fibroblasts adhesion and proliferation; tissue engineering application. | Esmail et al.[32] |

| | | | | | | | |
|---|---|---|---|---|---|---|---|
| | | | | respectively). | (complete wetting). | | |
| PHB/PHO, $CHCl_3$ | Randomly oriented fibers that mimic ECM; addition of PHO led to increase in blend fiber radius (from 336±98 to 744±462 nm for 1:0.25 and 1:1, respectively) | - | Decrease in stiffness upon addition of PHO (868±540kPa to 274±22kPa); matches articular cartilage. | | | Supports adhesion, migration, and synthetic activity of human articular cartilage chondrocytes; cartilage tissue engineering. | Ching et al.[35] |
| Combination of micro (PHB) and nanofibers (gelatin) crosslinked with genipin | Entangled network of micro (PHB, average diameter 1.21 μm) and nanofibers (gelatin, 0.22 μm); crosslinking with genipin increases the diameter of gel fibers to 0.338±0.193 μm and causes the change in surface roughness. | - | TS 0.78±0.35MPa YM 44.8±11.2 MPa ε 1.85±0.37% . | Increase in $\chi_c$ (12 to 19.1%). | Increase in WCA (0 to 38.7±6.1°). | Improved biocompatibility; great attachment of Balb/3T3 fibroblasts; no hemolytic activity; *in vivo* wound healing assay in rats has shown that gelatin containing dressing has induced almost complete wound closure; diabetic wound treatment. | Sanhueza et al.[36] |
| PHB/HA[i] nanocomposite immersed in metacrylated gelatin | Fiber diaameter 2±0.2 μm. | Improved thermal stability due to the HA incorporation. | Decrease in TS and E upon soaking in gelatin due to partial hydrolysis of PHA fibers. | - | - | Supports *in vitro* mineralization; adhesion, proliferation, and osteogenic phenotype of immobilized mouse calvarial preosteoblasts; Bone tissue engineering. | Sadat-Shojai et al.[37] |
| PHB, PHB/Curcumin, PHB/Quercetin, | Incorporation of curcumin caused decrease in fiber | - | - | Decrease in $\chi_c$ (31.53 vs. 24.28, and 21.22% for | - | *In vitro* antioxidative activity; biomedical application | Vilchez et al.[27] |

| | | | | | | | |
|---|---|---|---|---|---|---|---|
| | diameter (1.370±0.282 to 0.761±0.123 μm); quercetin - increase in diameter (to 1.803±0.557 μm) due to better solubility and more favorable Cur-PHB interactions. | | | PHB, PHB/Cur and PHB/Que, respectively). | | | |
| P4HB, P4HB/peptide analogue of fibroblast growth factor (GF), 19 % w/v, $CHCl_3$/DMSO | Addition of GF has reduced fiber diameter (6,9±0.02, to 5.0±0.05 to 4.8+-0.03 μm), for P4HB in $CHCl_3$, P4HB in $CHCl_3$/DMSO, and P4HB/GF/$CHCl_3$/DMSO, respectively; due to the increase in electrospinning solution conductivity. | A slight change in $T_m$ (56.0 to 54.3 to 55.2°C, for P4HB in $CHCl_3$, P4HB in $CHCl_3$/DMSO, and P4HB/GF/$CHCl_3$/DMSO, respectively); Thermal stability independent of processing conditions and GF incorporation. | Addition of GF has reduced TS (28 to15MPa) increased ε (360 to 730% for P4HB in CHCl3 vs. P4HB/GF in $CHCl_3$/DMSO). | - | Increase in WCA (103 to 121°). | *In vitro* wound healing assay showed that the scaffold supports fibroblast migration, adhesion, and proliferation; wound dressing. | Keridou et al.[28] |
| PHBV/OLE[j], PHB/PHOHD/OLE | Incorporation of OLE caused bead formation and fused regions in PHB/PHOHD/OLE fibers; mini reservoirs of polyphenols. | - | - | - | - | Burst release in first 30 min, 90% release in 6h; Immunomodulatory effect on human dermal keratinocytes, downregulation of proinflammatory cytokines; wound healing. | De la Ossa et al.[20] |
| PHB/cellulose acetate (CA), 5 wt%, | Decrease in composite fibers diameter upon | Increase in $T_g$ (12.6 to 36.5°C for PHB and | Decrease in TS (7.44±0.58 to | Decrease in the heat fusion of the crystalline phase | Decrease in WCA | Improved *in vitro* biodegradation; supports adhesion and | Zhijiang et al.[29] |

| | | | | | | | |
|---|---|---|---|---|---|---|---|
| $CHCl_3$/DMF | addition of CA (670±220 to 160±52 nm for PHB and PHB/CA 60/40, respectively). | PHB/CA 60/40), respectively) due to inhibited mobility of polymer chains (interactions with CA); decrease in $T_m$ (152.4 to 147.6°C for PHB and PHB/CA 60/40, respectively). | 4.52±0.34MPa), YM (867.5±201.2 to 806,9±168.2MPa) and ε (22.70±1.77 to 6.53±0.24%) for PHB and PHB/CA 60/40, respectively). | (72.4 to 25.8 J/g for PHB and PHB/CA 60/40, respectively). | (123.0±3.5 to 60.00±1.25 °) for PHB and PHB/CA 60/40, respectively). | proliferation of fibroblasts; wound dressing/tissue engineering. | |
| PHB/chitosan/bioglass, trifluoroacetic acid | Decrease in diameter due to increase in electrical conductivity of the spinning solution; presence of fused fiber regions. | - | Increase in TS (0.95±1.5 to 3,42±2.98 MPa) and E (109.9±0.05 to 210.96±0.09 MPa). | - | - | Supports differentiation of human exfoliated deciduous teeth stem cells to odontoblast-like cells and odontogenic phenotype; dentin tissue engineering. | Khoroushi et al.[19] |
| PHBV/chitosan/HA, 8% w/v, HFP | Decrease in nanocomposite fiber diameter (405±74 to 256±110 nm, for PHBV and PHBV/Ch/HA). | - | Decrease in TS (5.72+-0.42 to 3.55+-0.22 MPa), decrease in YM (151.26±1.61 to 101.93±4.52 MPa) and decrease in ε (31.81±2.45 to 4.4±0.08%). | - | Decrease in WCA (126.10±1.08 to 78.80±2.28 °). | Slow *in vitro* biodegradation (up to 60days); supports proliferation and osteogenic phenotype of human fetal osteoblasts; *in vitro* biomineralization; bone tissue engineering. | Zhang et al.[30] |
| PHBV, green solvents derived from levulinic acid (obtained from hemi-or cellulose fraction of biomass). | Ribbon-like fibers obtained using a mixture of formic acid and MEK (methyl ethyl ketone, a derivative of levulinic acid). | - | In agreement with the literature. | - | In agreement with the literature. | Supports adhesion and proliferation of adenocarcinoma human alveolar basal epithelial cells (A549)-*in vitro* model of lung endothelium; *in vitro* model of biological | Lapomarda et al.[21] |

barriers.

[a] 1,1,1,3,3,3-hexafluoro-2-propanol
[b] silver nanoparticles
[c] poly(D,L-lactic acid)
[d] dimethylformamide
[e] polyethylene oxide
[f] poly(L-lactide-*co*-$\varepsilon$-caprolactone)
[g] poly(3-hydroxyoctanoate-*co*-3-hydroyhexanoate)
[h] poly(3-hydroxyoctanoate-*co*-3-hydroydecanoate)
[i] hydroxyapatite
[j] olive leaf extract rich in polyphenols

## References:

[1] P. Kurtycz, E. Karwowska, T. Ciach, A. Olszyna, and A. Kunicki, "Biodegradable polylactide (PLA) fiber mats containing Al2O3-Ag nanopowder prepared by electrospinning technique — Antibacterial properties," *Fibers and Polymers*, vol. 14, no. 8, pp. 1248–1253, Aug. 2013, doi: 10.1007/s12221-013-1248-3.

[2] C. K. Yoon, B. K. Park, and W. Il Lee, "Characteristics of micro-glass bead/PLA porous composite prepared by electrospinning," *Advanced Composite Materials*, vol. 27, no. 2, pp. 183–193, Mar. 2018, doi: 10.1080/09243046.2017.1405508.

[3] M. Sabzi, L. Jiang, M. Atai, and I. Ghasemi, "PLA/sepiolite and PLA/calcium carbonate nanocomposites: A comparison study," *J Appl Polym Sci*, vol. 129, no. 4, pp. 1734–1744, Aug. 2013, doi: 10.1002/app.38866.

[4] S. Chung, M. P. Gamcsik, and M. W. King, "Novel scaffold design with multi-grooved PLA fibers," *Biomedical Materials*, vol. 6, no. 4, p. 045001, Aug. 2011, doi: 10.1088/1748-6041/6/4/045001.

[5] Y. Li *et al.*, "High-hydrophobic ZIF-8@PLA composite aerogel and application for oil-water separation," *Sep Purif Technol*, vol. 270, p. 118794, Sep. 2021, doi: 10.1016/j.seppur.2021.118794.

[6] I. Spiridon and C. E. Tanase, "Design, characterization and preliminary biological evaluation of new lignin-PLA biocomposites," *Int J Biol Macromol*, vol. 114, pp. 855–863, Jul. 2018, doi: 10.1016/j.ijbiomac.2018.03.140.

[7] Z. Wang, C. Zhao, and Z. Pan, "Porous bead-on-string poly(lactic acid) fibrous membranes for air filtration," *J Colloid Interface Sci*, vol. 441, pp. 121–129, Mar. 2015, doi: 10.1016/j.jcis.2014.11.041.

[8] L. Bai, L. Jia, Z. Yan, Z. Liu, and Y. Liu, "Plasma-etched electrospun nanofiber membrane as adsorbent for dye removal," *Chemical Engineering Research and Design*, vol. 132, pp. 445–451, Apr. 2018, doi: 10.1016/j.cherd.2018.01.046.

[9] C. Zhijiang, Z. Qin, S. Xianyou, and L. Yuanpei, "Zein/Poly(3-hydroxybutyrate-co-4-hydroxybutyrate) electrospun blend fiber scaffolds: Preparation, characterization and cytocompatibility," *Materials Science and Engineering: C*, vol. 71, pp. 797–806, Feb. 2017, doi: 10.1016/j.msec.2016.10.053.

[10] S. Steplin Paul Selvin, J. Lee, S. Kumar, N. Radhika, J. Princy Merlin, and I. Sharmila Lydia, "Photocatalytic degradation of rhodamine B using cysteine capped ZnO/P(3HB-co-3HHx) fiber under UV and visible light irradiation," *Reaction Kinetics, Mechanisms and Catalysis*, vol. 122, no. 1, pp. 671–684, Oct. 2017, doi: 10.1007/s11144-017-1232-9.

[11] C. Zhijiang, J. Jianru, Z. Qing, Z. Shiying, and G. Jie, "Preparation and characterization of novel Poly(3-Hydroxybutyrate-co-4-Hydroxybutyrate)/cellulose acetate composite fibers," *Mater Lett*, vol. 173, pp. 119–122, Jun. 2016, doi: 10.1016/j.matlet.2016.03.019.

[12] L. Chen *et al.*, "Multifunctional Electrospun Fabrics via Layer-by-Layer Electrostatic Assembly for Chemical and Biological Protection," *Chemistry of Materials*, vol. 22, no. 4, pp. 1429–1436, Feb. 2010, doi: 10.1021/cm902834a.

[13] P. Azari, R. Yahya, C. S. Wong, and S. N. Gan, "Improved processability of electrospun poly[(R)-3-hydroxybutyric acid] through blending with medium-chain length poly(3-hydroxyalkanoates) produced by *Pseudomonas putida* from oleic acid," *Materials Research Innovations*, vol. 18, no. sup6, pp. S6-345-S6-349, Dec. 2014, doi: 10.1179/1432891714Z.0000000001024.

[14] R. F. Canadas *et al.*, "Polyhydroxyalkanoates: Waste glycerol upgrade into electrospun fibrous scaffolds for stem cells culture," *Int J Biol Macromol*, vol. 71, pp. 131–140, Nov. 2014, doi: 10.1016/j.ijbiomac.2014.05.008.

[15] A. Wagner, V. Poursorkhabi, A. K. Mohanty, and M. Misra, "Analysis of Porous Electrospun Fibers from Poly( <scp>l</scp> -lactic acid)/Poly(3-hydroxybutyrate- *co* -3-hydroxyvalerate) Blends," *ACS Sustain Chem Eng*, vol. 2, no. 8, pp. 1976–1982, Aug. 2014, doi: 10.1021/sc5000495.

[16] O. Vega-Castro *et al.*, "Characterization and Production of a Polyhydroxyalkanoate from Cassava Peel Waste: Manufacture of Biopolymer Microfibers by Electrospinning," *J Polym Environ*, vol. 29, no. 1, pp. 187–200, Jan. 2021, doi: 10.1007/s10924-020-01861-1.

[17] T. H. Ying *et al.*, "Scaffolds from electrospun polyhydroxyalkanoate copolymers: Fabrication, characterization, bioabsorption and tissue response," *Biomaterials*, vol. 29, no. 10, pp. 1307–1317, Apr. 2008, doi: 10.1016/j.biomaterials.2007.11.031.

[18] W. Li, N. Cicek, D. B. Levin, and S. Liu, "Enabling electrospinning of medium-chain length polyhydroxyalkanoates (PHAs) by blending with short-chain length PHAs," *International Journal of Polymeric Materials and Polymeric Biomaterials*, vol. 68, no. 9, pp. 499–509, Jun. 2019, doi: 10.1080/00914037.2018.1466136.

[19] M. Khoroushi, M. R. Foroughi, S. Karbasi, B. Hashemibeni, and A. A. Khademi, "Effect of Polyhydroxybutyrate/Chitosan/Bioglass nanofiber scaffold on proliferation and differentiation of stem cells from human exfoliated deciduous teeth into odontoblast-like cells," *Materials Science and Engineering: C*, vol. 89, pp. 128–139, Aug. 2018, doi: 10.1016/j.msec.2018.03.028.

[20] J. G. De la Ossa *et al.*, "Immunomodulatory Activity of Electrospun Polyhydroxyalkanoate Fiber Scaffolds Incorporating Olive Leaf Extract," *Applied Sciences*, vol. 11, no. 9, p. 4006, Apr. 2021, doi: 10.3390/app11094006.

[21] A. Lapomarda *et al.*, "Valorization of a Levulinic Acid Platform through Electrospinning of Polyhydroxyalkanoate-Based Fibrous Membranes for In Vitro Modeling of Biological Barriers," *ACS Appl Polym Mater*, vol. 4, no. 8, pp. 5872–5881, Aug. 2022, doi: 10.1021/acsapm.2c00721.

[22] K. Sombatmankhong, O. Suwantong, S. Waleetorncheepsawat, and P. Supaphol, "Electrospun fiber mats of poly(3-hydroxybutyrate), poly(3-hydroxybutyrate-co-3-hydroxyvalerate), and their blends," *J Polym Sci B Polym Phys*, vol. 44, no. 19, pp. 2923–2933, Oct. 2006, doi: 10.1002/polb.20915.

[23] A. Bianco, M. Calderone, and I. Cacciotti, “Electrospun PHBV/PEO co-solution blends: Microstructure, thermal and mechanical properties,” *Materials Science and Engineering: C*, vol. 33, no. 3, pp. 1067–1077, Apr. 2013, doi: 10.1016/j.msec.2012.11.030.

[24] D. Daranarong, R. T. H. Chan, N. S. Wanandy, R. Molloy, W. Punyodom, and L. J. R. Foster, “Electrospun Polyhydroxybutyrate and Poly(L-lactide- *co* - *ε* -caprolactone) Composites as Nanofibrous Scaffolds,” *Biomed Res Int*, vol. 2014, pp. 1–12, 2014, doi: 10.1155/2014/741408.

[25] Z.-C. Xing, S.-J. Han, Y.-S. Shin, and I.-K. Kang, “Fabrication of Biodegradable Polyester Nanocomposites by Electrospinning for Tissue Engineering,” *J Nanomater*, vol. 2011, pp. 1–18, 2011, doi: 10.1155/2011/929378.

[26] C. Lei, H. Zhu, J. Li, J. Li, X. Feng, and J. Chen, “Preparation and characterization of polyhydroxybutyrate- *co* -hydroxyvalerate/silk fibroin nanofibrous scaffolds for skin tissue engineering,” *Polym Eng Sci*, vol. 55, no. 4, pp. 907–916, Apr. 2015, doi: 10.1002/pen.23958.

[27] A. Vilchez, F. Acevedo, M. Cea, M. Seeger, and R. Navia, “Development and thermochemical characterization of an antioxidant material based on polyhydroxybutyrate electrospun microfibers,” *Int J Biol Macromol*, vol. 183, pp. 772–780, Jul. 2021, doi: 10.1016/j.ijbiomac.2021.05.002.

[28] I. Keridou, L. Franco, J. C. Martínez, P. Turon, L. J. Del Valle, and J. Puiggalí, “Electrospun scaffolds for wound healing applications from poly(4-hydroxybutyrate): A biobased and biodegradable linear polymer with high elastomeric properties,” *J Appl Polym Sci*, vol. 139, no. 1, p. 51447, Jan. 2022, doi: 10.1002/app.51447.

[29] C. Zhijiang, X. Yi, Y. Haizheng, J. Jia, and Y. Liu, “Poly(hydroxybutyrate)/cellulose acetate blend nanofiber scaffolds: Preparation, characterization and cytocompatibility,” *Materials Science and Engineering: C*, vol. 58, pp. 757–767, Jan. 2016, doi: 10.1016/j.msec.2015.09.048.

[30] S. Zhang, M. P. Prabhakaran, X. Qin, and S. Ramakrishna, “Biocomposite scaffolds for bone regeneration: Role of chitosan and hydroxyapatite within poly-3-hydroxybutyrate-co-3-hydroxyvalerate on mechanical properties and in vitro evaluation,” *J Mech Behav Biomed Mater*, vol. 51, pp. 88–98, Nov. 2015, doi: 10.1016/j.jmbbm.2015.06.032.

[31] M.-L. Cheng, P.-Y. Chen, C.-H. Lan, and Y.-M. Sun, “Structure, mechanical properties and degradation behaviors of the electrospun fibrous blends of PHBHHx/PDLLA,” *Polymer (Guildf)*, vol. 52, no. 6, pp. 1391–1401, Mar. 2011, doi: 10.1016/j.polymer.2011.01.039.

[32] A. Esmail *et al.*, “Oxygen Plasma Treated-Electrospun Polyhydroxyalkanoate Scaffolds for Hydrophilicity Improvement and Cell Adhesion,” *Polymers (Basel)*, vol. 13, no. 7, p. 1056, Mar. 2021, doi: 10.3390/polym13071056.

[33] J. Yuan *et al.*, “Novel wound dressing based on nanofibrous PHBV-keratin mats,” *J Tissue Eng Regen Med*, vol. 9, no. 9, pp. 1027–1035, Sep. 2015, doi: 10.1002/term.1653.

[34] K. Sudesh, Y.-F. Lee, N. Sridewi, and S. Ramanathan, “The Influence of Electrospinning Parameters and Drug Loading on Polyhydroxyalkanoate (PHA) Nanofibers for Drug Delivery,” *Int J Biotechnol Wellness Ind*, vol. 4, no. 4, pp. 103–113, Jan. 2016, doi: 10.6000/1927-3037.2015.04.04.1.

[35] K. Y. Ching *et al.*, "Nanofibrous poly(3-hydroxybutyrate)/poly(3-hydroxyoctanoate) scaffolds provide a functional microenvironment for cartilage repair," *J Biomater Appl*, vol. 31, no. 1, pp. 77–91, Jul. 2016, doi: 10.1177/0885328216639749.

[36] C. Sanhueza *et al.*, "One-step electrospun scaffold of dual-sized gelatin/poly-3-hydroxybutyrate nano/microfibers for skin regeneration in diabetic wound," *Materials Science and Engineering: C*, vol. 119, p. 111602, Feb. 2021, doi: 10.1016/j.msec.2020.111602.

[37] M. Sadat-Shojai, M.-T. Khorasani, and A. Jamshidi, "A new strategy for fabrication of bone scaffolds using electrospun nano-HAp/PHB fibers and protein hydrogels," *Chemical Engineering Journal*, vol. 289, pp. 38–47, Apr. 2016, doi: 10.1016/j.cej.2015.12.079.
